# Endothelial SLC38A2 controls brain D-serine homeostasis and synapse development

**DOI:** 10.1016/j.isci.2026.117487

**Published:** 2026-09-09

**Authors:** Akshay K. Tiwari, Ankita Lahkar, Clara Sajrawi, Yara Hussein, Adam Majcher, Stephane Bonelli, Bella Agranovich, Ifat Abramovich, Shani Stern, Farrukh A. Chaudhry, Thorsten Hornemann, Inna Radzishevsky, Herman Wolosker

**Affiliations:** 1 Technion-Israel Institute of Technology, Ruth and Bruce Rappaport Faculty of Medicine, Department of Biochemistry, Haifa 3525433, Israel; 2 Sagol Department of Neurobiology, Faculty of Natural Sciences, University of Haifa, Haifa 3103301, Israel; 3 Institute of Clinical Chemistry, University of Zurich and University Hospital Zurich, 8091 Zurich, Switzerland; 4 Technion-Israel Institute of Technology, Ruth and Bruce Rappaport Faculty of Medicine, Laura and Isaac Perlmutter Metabolomics Center, Haifa 3525433, Israel; 5 The Institute of Basic Medical Sciences, University of Oslo, Blindern, 0317 Oslo, Norway

**Keywords:** serine, blood-brain barrier, D-serine, amino acid transport

## Abstract

L-serine is a precursor for the synthesis of the neuromodulator D-serine, glycine, and lipids. Contrary to the prevailing view that brain L-serine is exclusively derived from *de novo* synthesis, we demonstrate that dietary L-serine is required to maintain brain L-serine levels and prevents the formation of neurotoxic 1-deoxysphingolipids. We identified SLC38A2 as an L-serine transporter at the blood-brain barrier (BBB) that mediates the influx of circulating L-serine to the brain. Endothelial-selective Slc38a2 conditional knockout (*Slc38a2*-cKO) decreased *in vivo* blood-to-brain L-serine influx and selectively lowered hippocampal L-serine and D-serine in mouse pups. Immunoelectron microscopy demonstrated that SLC38A2 is localized to both the luminal and abluminal endothelial membranes. *Slc38a2*-cKO pups exhibited ultrastructural defects at excitatory synapses, including reduced synaptic vesicle cluster size and decreased postsynaptic density area. SLC38A2 inhibition may be beneficial for limiting D-serine accumulation in conditions with pathologically elevated L- or D-serine levels, such as traumatic brain injury and epilepsy.

## Highlights

• Dietary L-serine controls the formation of neurotoxic 1-deoxysphingolipids and D-serine
• SLC38A2 mediates blood-to-brain L-serine transport across the blood-brain barrier
• Deletion of SLC38A2 reduces brain L-serine and the neuromodulator D-serine
• Loss of SLC38A2 causes ultrastructural defects at excitatory synapses

## Graphical abstract

**Figure:**
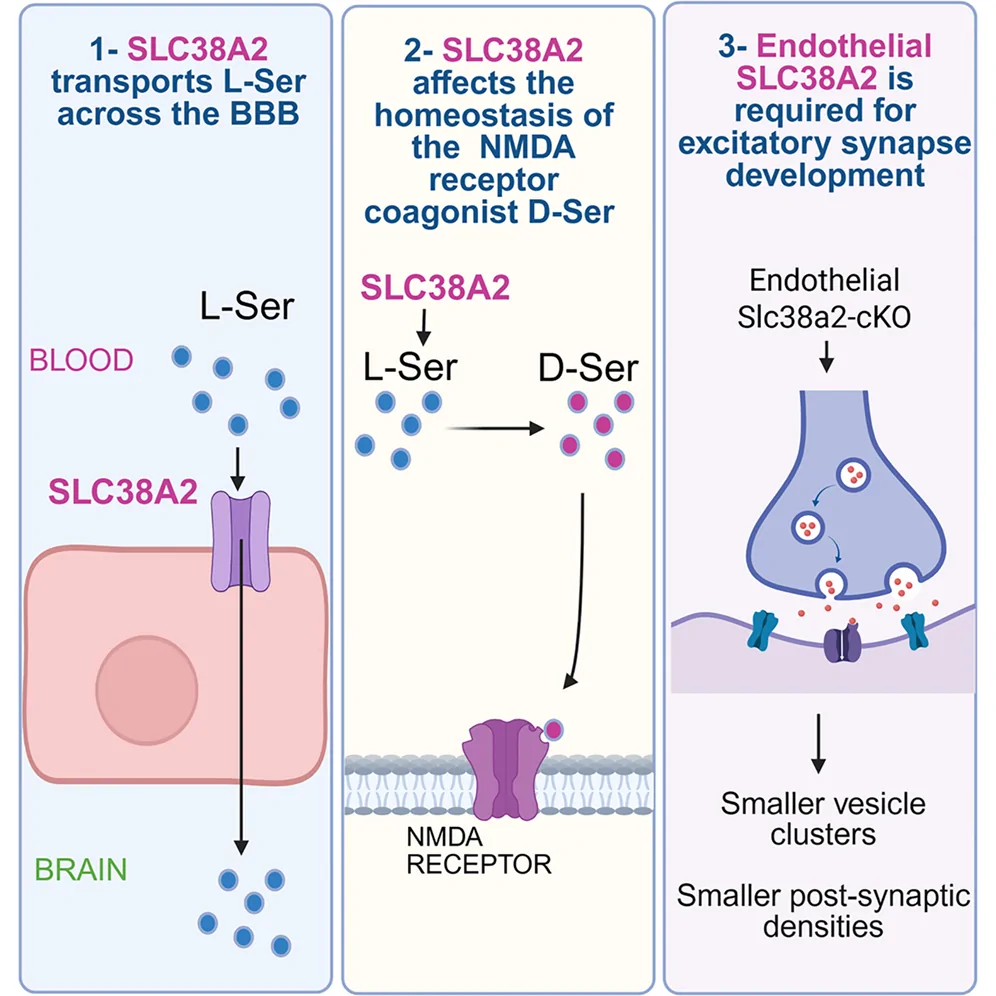

## Teaser

Molecular neuroscience; Cellular neuroscience; Metabolic flux analysis

## Introduction

The blood-brain barrier (BBB) mediates a developmental influx of amino acids from the circulation into the brain, a process that is essential for brain growth and synapse formation.^1^^,^^2^^,^^3^ The major transporter for essential amino acids at the BBB is LAT1 (SLC7A5),^4^ whereas cationic amino acids are transported primarily by CAT-1 (SLC7A1), localized at the luminal membrane of endothelial cells.^5^ In contrast, the transport of non-essential amino acids across the BBB has only recently begun to be understood. For example, serine and glutamine transport through the BBB is critical for neurodevelopment, and mutations or targeted deletion of their transporters cause microcephaly and other neurodevelopmental abnormalities.^1^^,^^2^^,^^3^^,^^5^^,^^6^^,^^7^

Until recently, circulating serine was not considered a major contributor to brain metabolism. In adult mice, knockout of Phgdh, which encodes a key enzyme in *de novo* L-serine synthesis in the brain, markedly reduces brain L-serine levels,^8^ supporting the view that brain serine is derived predominantly from local synthesis. However, recent studies indicate that early postnatal mouse pups show a greater influx of blood-derived L-serine into the brain than older animals. These findings suggest that blood-to-brain transport of L-serine is developmentally regulated and may be required to support brain metabolism during early life. We have shown that deletion of endothelial serine transporters SLC38A5 and SLC1A4, which are transiently enriched in brain capillaries of young pups, lowers the influx of L-serine through the BBB and reduces brain D-serine, a physiological co-agonist of NMDA receptors (NMDARs).^1^^,^^3^ These changes are accompanied by synaptic and metabolic abnormalities, including the accumulation of toxic 1-deoxysphingolipids (1-deoxySLs). These atypical sphingolipids (SLs) are produced when brain L-serine levels fall, and serine palmitoyltransferase increasingly uses alanine instead of serine, generating 1-deoxysphinganine, the precursor of all 1-deoxySLs, including deoxydihydroceramides.^9^^,^^10^ Nevertheless, given their transient enrichment at the BBB in young mice, the contributions of SLC1A4 and SLC38A5 to brain serine and SL metabolism are restricted to mouse pups. SLC1A4 and SLC1A5 also handle additional substrates, including threonine, and may therefore influence brain function through different mechanisms.

SLC38A2 (SNAT2) is a broad-spectrum neutral amino acid transporter of the system A family that mediates the *in vitro* transport of serine, alanine, methionine, cysteine, asparagine, proline, and glutamine.^11^^,^^12^^,^^13^ SLC38A2 expression is induced by amino acid deprivation, which increases SLC38A2 transcription and protein stability.^14^^,^^15^ Transcription of SLC38A2 in hepatoma cells is strongly enhanced by ATF4 and contributes to amino acid-dependent SLC32A2 regulation.^16^ Insulin stimulates SLC38A2 activity by recruiting the transporter to the plasma membrane of muscle cells.^17^^,^^18^ Adenoviral-mediated hepatic expression of SLC38A2 activates the intracellular target of rapamycin-1 (mTORC1) signaling pathway, changes liver metabolism, and increases serum triglycerides.^19^ In the brain, SLC38A2 is expressed at glutamatergic synapses, where it is proposed to supply neurons with glutamine for glutamate synthesis.^20^^,^^21^ On the other hand, Grewal et al.^22^ did not find an effect of SLC32A2 induction or repression on excitatory transmission in cultured neurons. SLC38A2 is also expressed in the neonatal endothelium of brain capillaries that compose the BBB,^23^ but its physiological function and the exact *in vivo* substrates of this transporter remain unknown.

Despite the prevailing view that all L-serine is synthesized in the brain by the phosphorylated pathway,^24^ we show here that dietary L-serine is essential for brain D-serine synthesis, SL homeostasis, and gene expression, supporting blood-to-brain L-serine transport across the BBB. Using endothelial-specific Slc38a2 conditional knockout (cKO) mice, we identify SLC38A2 as a physiologic L-serine transporter at the BBB. We further find that SLC38A2 is localized to both the luminal and abluminal membranes of endothelial cells, where it performs distinct developmental functions. The luminal SLC38A2 operates as a selective L-serine influx transporter in preweaning mice. In contrast, juvenile and adult *Slc38a2*-cKO mice show elevated brain levels of glutamine and proline, which implies that abluminal SLC38A2 may mediate equilibrative efflux of brain SLC38A2 substrates. Together, these findings identify endothelial SLC38A2 as a novel regulator of brain amino acid homeostasis via its BBB expression, which contributes to glutamatergic synapse development.

## Results

### Dietary L-serine is essential to maintain brain L-serine and D-serine

**Figure 1:**
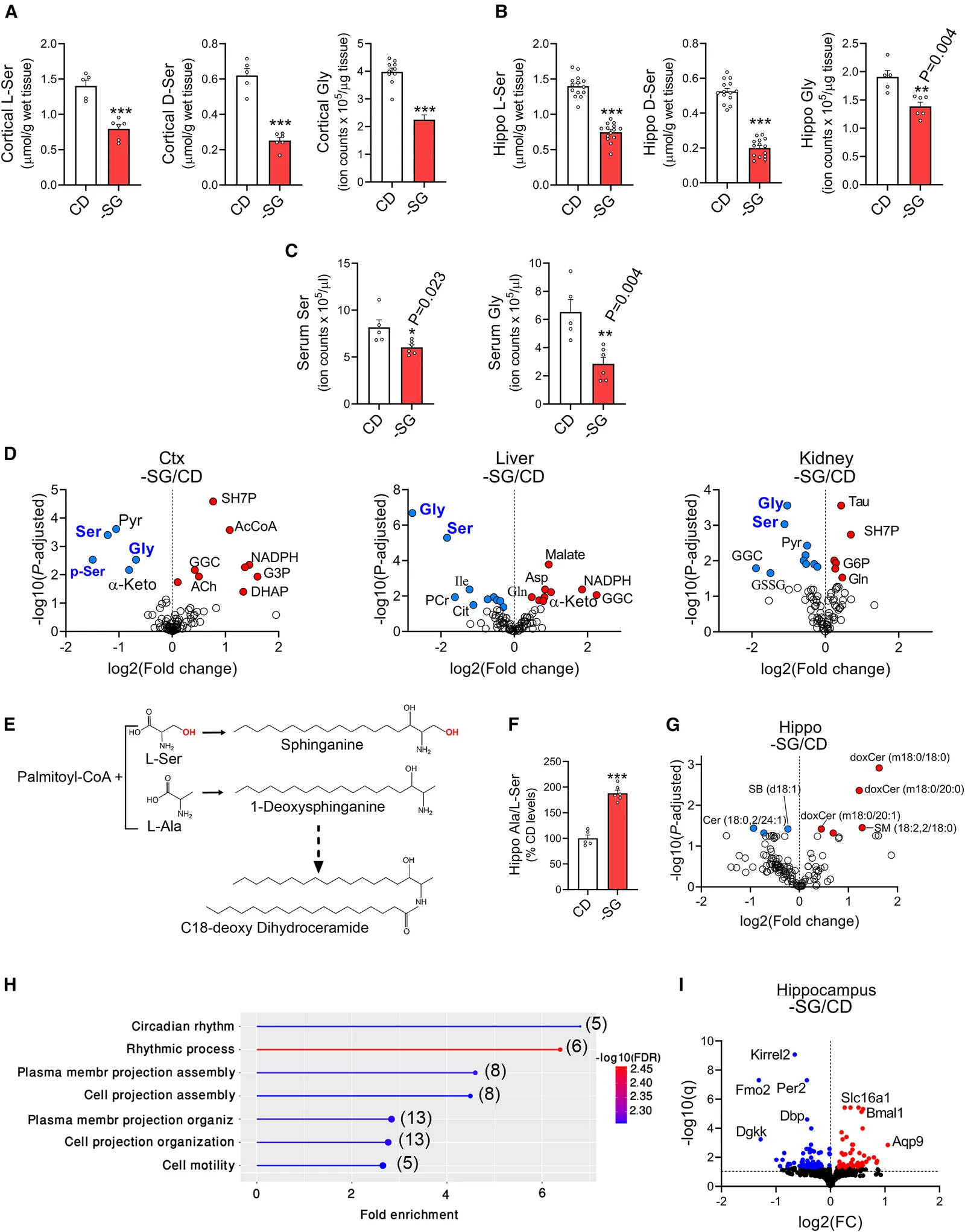
Dietary L-serine regulates the metabolome, sphingolipidome, and gene expression across multiple tissues (A) Three-week-old mice were fed either an amino acid-defined control diet (CD) or a serine- and glycine-restricted diet (-SG) for 10 days. Mice fed the -SG diet exhibited reduced cortical levels of L-serine, D-serine, and glycine. The data are mean ± SEM of 5–14 mice per group; Student’s *t* test. (B) Hippocampal levels of L-serine, D-serine, and glycine in -SG-fed mice. The data are mean ± SEM of 5–10 mice per group; Student’s *t* test. (C) -SG diet reduced serum total serine and glycine levels. The data are mean ± SEM of 5–6 mice per group; Student’s *t* test. (D) Volcano plots from targeted metabolomic analysis of the cerebral cortex, liver, and kidneys of mice fed the -SG diet compared with CD controls (*n* = 10 mice per group). Significantly changed metabolites with a false discovery rate (FDR) of less than 0.05 are depicted in color. (E) Schematic of serine palmitoyltransferase reactions with L-serine or L-alanine, leading to the formation of canonical sphingolipids or 1-deoxysphingolipids, respectively. (F) -SG diet increased the alanine-to-total serine ratio in the hippocampus relative to CD controls. The data are mean ± SEM of 5–6 mice per group; Student’s *t* test. (G) Volcano plot of hippocampal sphingolipidomic analysis showing increased levels of several 1-deoxysphingolipid species (*n* = 10 mice per group). Significantly changed metabolites with an FDR < 0.05 are depicted in color. (H) Gene Ontology (GO) enrichment analysis of bulk RNA-seq data from -SG-fed mice relative to CD-fed controls (*n* = 5 mice per group). (I) Differential gene expression analysis between -SG- and CD-fed mice. (*n* = 5 mice per group) (FDR < 0.05).

L-Serine is a non-essential amino acid generally thought to be synthesized entirely in the brain, without contribution from circulating or dietary L-serine.^8^ However, deletions or mutations in serine transporters expressed in endothelial cells suggest that L-serine may be imported through the BBB.^1^^,^^3^ Despite this, there has been no direct evidence that dietary L-serine contributes to brain pools of L-serine or its derivative D-serine. To evaluate this possibility, 3-week-old mice were fed a serine/glycine-deficient diet (-SG) for 10 days and compared with littermates receiving an amino acid-defined control diet (CD). To minimize replenishment of L-serine through interconversion with glycine, the -SG diet was also restricted in glycine. Analysis of serine enantiomers by high-performance liquid chromatography (HPLC) revealed that the -SG diet reduced hippocampal L-serine by 45% and D-serine by 60% (Figure 1A). A similar decrease was observed in the cerebral cortex (Figure 1B). Because D-serine is synthesized in the brain from L-serine by serine racemase^25^ these findings indicate that dietary L-serine is a major determinant of brain D-serine levels. Glycine levels were also reduced, although to a lesser extent (28%). Consistent with these changes, serum total serine and glycine levels were significantly decreased in mice fed the -SG diet, confirming that dietary restriction lowers the availability of these amino acids to the brain (Figure 1C).

We next examined whether feeding the -SG diet alters central carbon metabolism using targeted metabolomics. Principal component analysis (PCA) clearly separated metabolites of the cerebral cortex of -SG-fed mice from those fed a CD (Figure S1A), indicating a distinct metabolic profile. In addition to the reductions in total serine and glycine, the serine precursor L-phosphoserine was decreased, as were pyruvate and α-ketoglutarate, in the cortex of mice fed the -SG diet (Figure 1D; Table S1). Several metabolites were elevated, including sedoheptulose 7-phosphate, acetyl-CoA, glycerol 3-phosphate, NADPH, gamma-glutamylcysteine, and dihydroxyacetone phosphate, consistent with substantial metabolic reprogramming.

The metabolic profiles of the livers and kidneys from -SG-fed mice were also clearly separated from those of CD-fed controls by PCA (Figures S1B and S1C). Although each tissue displayed a distinct metabolic signature (Figure 1D; Table S1), total serine and glycine were the only metabolites altered across all tissues analyzed (Figure S1D).

### Dietary serine restriction alters sphingolipid metabolism and gene expression

Serine palmitoyltransferase catalyzes the first step in SL synthesis by condensing palmitoyl-CoA with serine (Figure 1E). When serine availability is limited, however, the enzyme instead uses alanine, generating 1-deoxysphinganine, the precursor of more complex 1-deoxySLs. These atypical SLs are cytotoxic and are a hallmark of serine deficiency.^1^ 1-DeoxySLs also accumulate in hereditary sensory and autonomic neuropathy type 1 and diabetic neuropathy, where they contribute to peripheral nerve damage.^10^^,^^26^ Mice fed the -SG diet exhibited a 2-fold increase in the brain Ala/Ser ratio (Figure 1F). Consistent with this shift, sphingolipidomic analysis of more than one hundred SL species revealed the accumulation of multiple 1-deoxySL species, such as 1-deoxydihydroceramides in the hippocampus (Figure 1G; Table S2). We confirmed that the hippocampus exhibits a decrease in serine by metabolomics analysis in another cohort (Figure S2 and Table S1). Changes in 1-deoxySLs were also detected in the liver and kidneys (Figure S3).

Mice fed the -SG diet also displayed altered gene expression in the hippocampus, most prominently a downregulation of circadian rhythm genes (Figures 1H and 1I; Table S3), including Bmal1, Cry1, Per2, Dbp, and Nfil3. The -SG diet also affected the expression of several endothelium-enriched or endothelium-associated genes, including *Cldn5*, *Mfsd2a*, *Pecam1*, *Slc16a1*, *Mmp14*, and *Sash1* (Table S5).

### Identification of SLC38A2 as a physiologic L-serine transporter at the BBB

**Figure 2:**
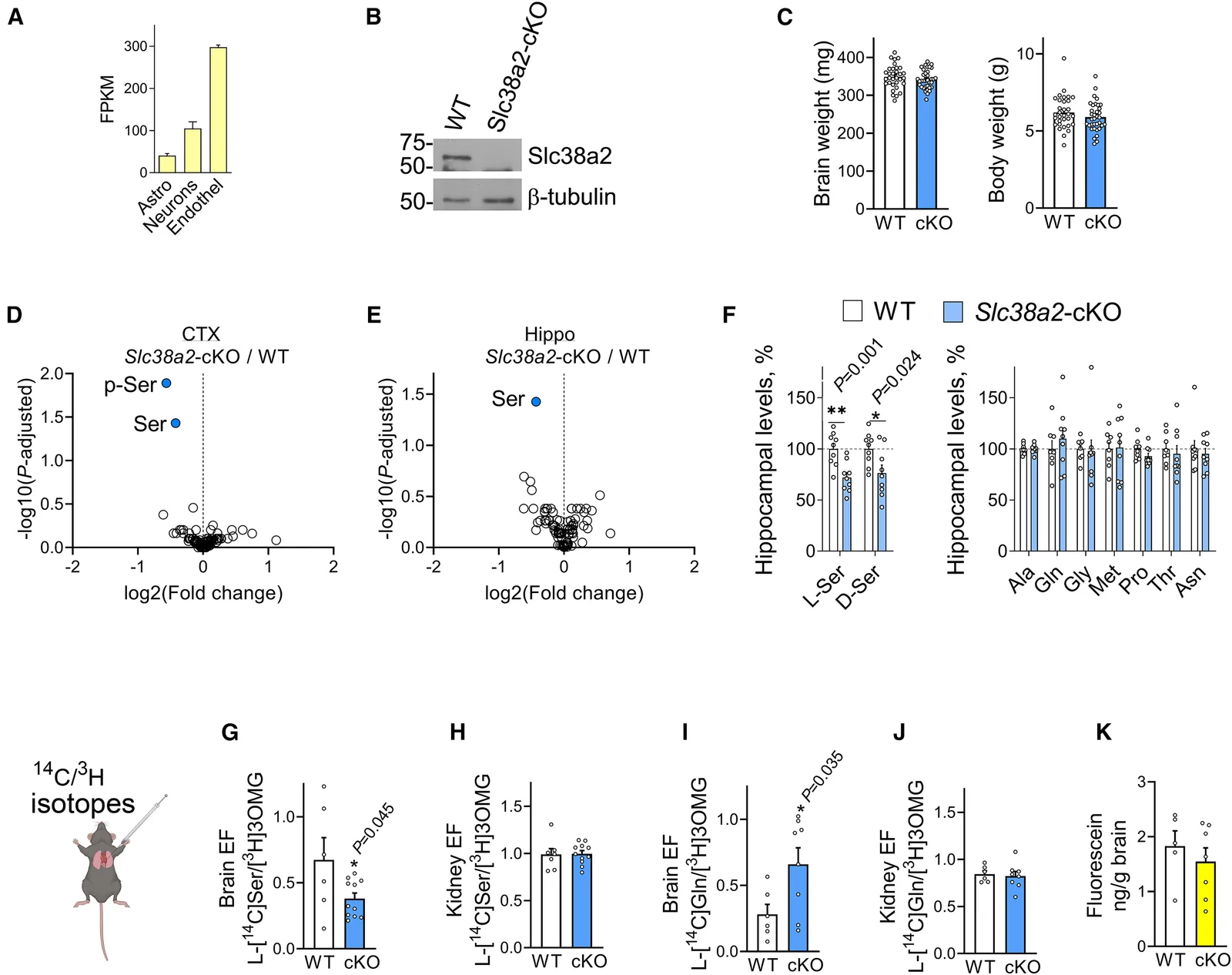
SLC38A2 is a physiological L-serine transporter at the BBB and is essential for brain serine metabolism (A) RNA-seq analysis comparing *Slc38a2* expression in endothelial cells, neurons, and astrocytes from a previously published dataset.^28^ FPKM, fragments per kilobase of exon per million mapped fragments. (B) Representative western blot showing Slc38a2 deletion in purified brain microvessels. The lower image shows β-tubulin as a loading control. (C) *Slc38a2*-cKO does not alter brain or body weight in mice. The data are mean ± SEM of 33–35 mice/group. Student’s *t* test. (D) Volcano plot showing altered cortical metabolites in *Slc38a2*-cKO mice (*n* = 8 mice/group). p-Ser, phosphoserine. Significantly changed metabolites with an FDR < 0.05 are depicted in color. (E) Volcano plot showing altered Ser and hippocampal metabolites in Slc38a2-cKO mice (*n* = 8 mice/group). Significantly changed metabolites having a false discovery rate (FDR) of less than 0.05 are depicted in color. (F) Left graph, enantiomeric HPLC separation showing reduced hippocampal L- and D-serine levels in *Slc38a2*-cKO mice. Absolute values of L-serine are (in μmol/g wet tissue, average ± SEM) 1.43 ± 0.08 (WT) and 1.03 ± 0.07 (Slc38a2-cKO). Absolute values of D-serine are 0.30 ± 0.02 (WT) and 0.23 ± 0.02 (Slc38a2-cKO). The right graph shows that Slc38a2-cKO has no effect on other amino acid substrates measured by LC-MS. The data are mean ± SEM of 8–9 mice/group; Student’s *t* test. (G) Reduced brain extraction fraction (EF) of intracardially injected L-[^14^C]serine in *Slc38a2*-cKO mice. The data were normalized by the EF of 3-O-Methylglucose (3OMG). The data are mean ± SEM of 6–11 mice/group; Student’s *t* test. (H) Unchanged EF of intracardially injected L-[^14^C]serine in the kidneys of *Slc38a2*-cKO mice. The data are mean ± SEM of 6–11 mice/group; Student’s *t* test. (I) Increased EF of intracardially injected L-[^14^C]glutamine in the brains of *Slc38a2*-cKO mice. The data are mean ± SEM of 6–11 mice/group; Student’s *t* test. (J) Unchanged EF of intracardially injected L-[^14^C]glutamine in the kidneys of *Slc38a2*-cKO mice. The data are mean ± SEM of 6–11 mice/group; Student’s *t* test. (K) Fluorescein brain penetration is unchanged in *Slc38a2*-cKO mice. The data are mean ± SEM of 6–11 mice/group; Student’s *t* test.

Given the marked effects of the -SG diet on brain L- and D-serine levels, we sought to identify and delete an endothelial transporter that mediates selective L-serine transport across the BBB. The two known endothelial serine transporters have a limited contribution to brain L-serine. Deletion of SLC38A5 decreased brain L-serine by 15%–20%,^3^ while conditional deletion of SLC1A4 in endothelial cells led to a 10% decrease in brain L-serine.^1^ These findings indicate the likely presence of at least one additional serine transporter at the BBB to account for the effects of dietary serine restriction. Our previous study with system A transporters demonstrated that SLC32A2 exhibits the highest efficiency in transporting L-serine *in vitro* compared with its canonical substrate, glutamine.^27^ Using a published RNA sequencing (RNA-seq) dataset^28^ and analyzing a human brain microvessel proteomics database,^23^ we found that the SLC38A2 transporter is also highly expressed in endothelial cells (Figure 2A). We deleted SLC38A2 in brain endothelial cells by crossing *Slc38a2* ^fl/fl^ with Tie2-Cre (*Slc38a2*-cKO). Western blot analysis of purified brain vessels confirmed the successful deletion of SLC38A2 (Figure 2B). At postnatal day 11 (P11), Slc38a2-cKO pups showed no changes in brain or body weight (Figure 2C). Since SLC38A2 expression is highest in the early developmental period,^23^ subsequent experiments were conducted using P11 pups.

Metabolic profiles of the cortex (Figure 2D; Table S4) and hippocampus (Figure 2E; Table S4) revealed a most selective decrease in the steady-state levels of total serine in *Slc38a2*-cKO pups. As observed in the cortex of -SG-fed mice, the serine precursor phosphoserine was also decreased in the cortex of *Slc38a2*-cKO pups (Figure 2D). No other metabolite or SLC38A2 substrate was affected, indicating a high selectivity for serine (Table S4). HPLC analysis of serine enantiomers revealed parallel reductions in hippocampal L-serine and D-serine in *Slc38a2*-cKO pups, but no changes in other SLC38A2 substrates measured by liquid chromatography-mass spectrometry (LC-MS) (Figure 2F). No change in total serine or other common metabolites was detected in the serum, suggesting brain-specific effects (Figure S4 and Table S4).

### Transport of L-serine across the BBB by SLC38A2

We next asked whether *Slc38a2*-cKO affects the *in vivo* import of L-serine across the BBB. We co-injected L-[^14^C]serine with [^3^H]3-O-methylglucose (3OMG) transcardiacally, and after 10 s, the intravascular isotopes were removed by brief perfusion with ice-cold PBS. The serine extraction fraction (EF) in the brain and kidneys was normalized by the concomitant uptake of 3OMG. Using this approach, we found that *Slc38a2*-cKO pups exhibited a 45% reduction in brain serine uptake (Figure 2G). In contrast, the serine EF in the kidneys was unchanged (Figure 2H), indicating that the effect of Slc38a2 deletion is specific to the brain.

Since SLC38A2 also uses glutamine as a substrate *in vitro*,^29^ we evaluated glutamine EF in the brain and kidneys. In contrast to L-serine, the brain EF of L-glutamine was more than 2-fold higher in *Slc38a2*-cKO pups (Figure 2I). The effect was brain-specific, as glutamine EF was unchanged in the kidneys (Figure 2J). These findings suggest that glutamine transport by SLC38A2 occurs in the opposite direction to that of serine. Despite the increased glutamine EF, steady-state brain glutamine levels were unchanged in *Slc38a2*-cKO pups (Figures 2D–2F), indicating that the higher glutamine influx does not measurably contribute to the brain steady-state levels of this substrate at this age.

Fluorescein permeability was unchanged in the *Slc38a2*-cKO, ruling out BBB disruption by the knockout (Figure 2K).

### Subcellular localization of SLC38A2 in endothelial cells

**Figure 3:**
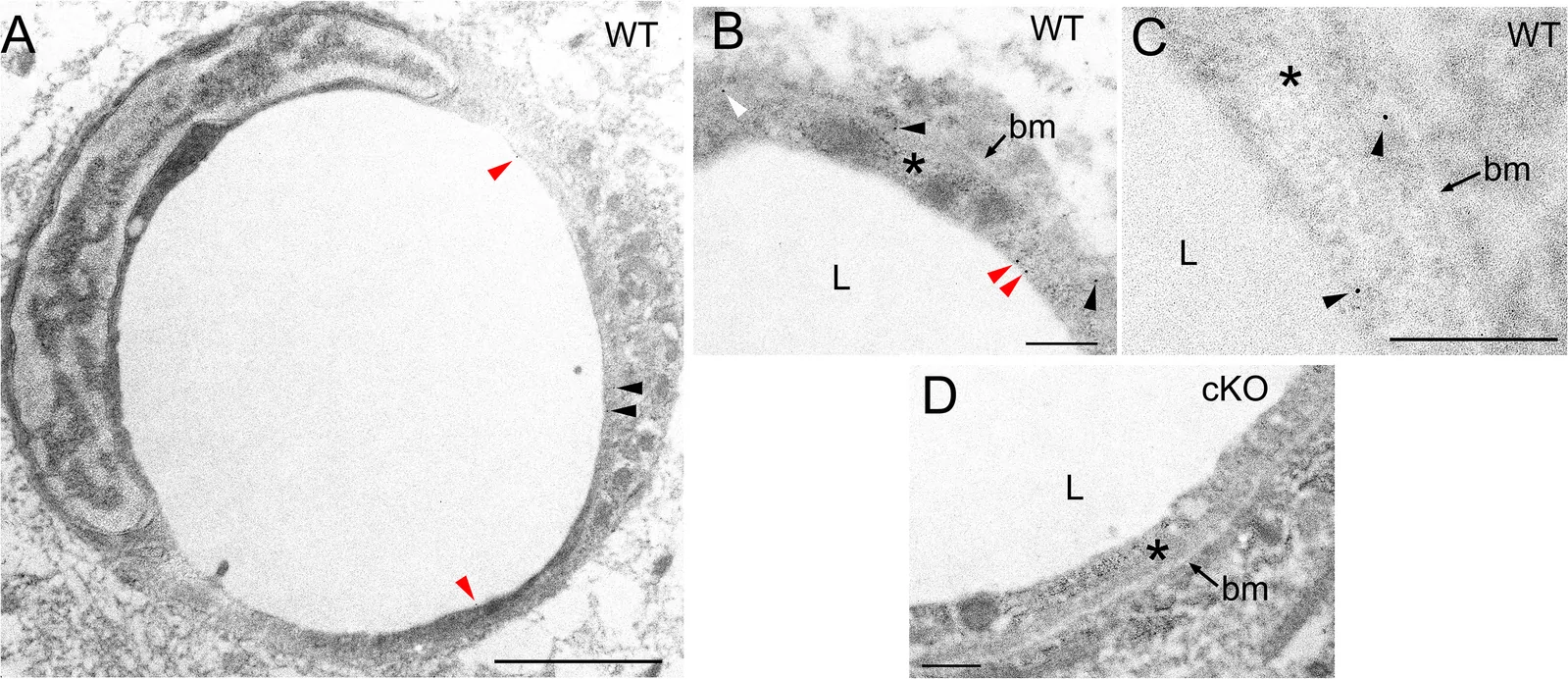
Presence of SLC38A2 at the luminal and abluminal membranes of endothelial cells (A) Representative electron micrograph of SLC38A2 labeled by colloidal gold at the luminal (red arrowheads) and intracellular (black arrowheads) locations in endothelial cells of the neocortex of P11 mice. Scale bars, 2 μm. (B) Representative SLC38A2 immunoreactivity in luminal (red arrowheads) and abluminal (white arrowheads) membranes. Intracellular labeling in an endothelial cell (∗) is depicted by black arrowheads. bm, basement membrane. Scale bars, 0.5 μm. (C) Representative intracellular colloidal gold immunoreactivity (black arrowheads) in an endothelial cell (∗). Scale bars, 0.5 μm. (D) Representative electron micrograph of *Slc38a2*-cKO mice showing lack of colloidal gold labeling in the endothelial cell. Scale bars, 1 μm.

A previous study using membrane vesicle preparations suggested that neutral amino acid transport activity is exclusively present at the abluminal membrane of brain endothelial cells.^30^ To directly evaluate SLC38A2 subcellular localization in brain capillaries, we carried out immunoelectron microscopy and found that gold particles recognizing SLC38A2 decorated both the luminal and abluminal membranes of endothelial cells (Figures 3A–3C). There was also some intracellular labeling. No immunoreactivity was detected in *Slc38a2*-cKO capillaries, confirming antibody specificity (Figure 3D).

### Metabolic role of endothelial SLC38A2

To determine how endothelial Slc38a2 deletion affects brain serine metabolism, we co-injected L-serine^13^C_1_^15^N_1_ and glucose^13^C_6_ in P11 pups to verify the utilization of serine and its concomitant biosynthesis from glucose. To prevent the conversion of L-serine into glycine in peripheral tissues, pups were pretreated with Shin1, a non-brain-penetrant inhibitor of serine hydroxymethyltransferase 1 and 2 (SHMT1 AND SHMT2).^3^^,^^31^

**Figure 4:**
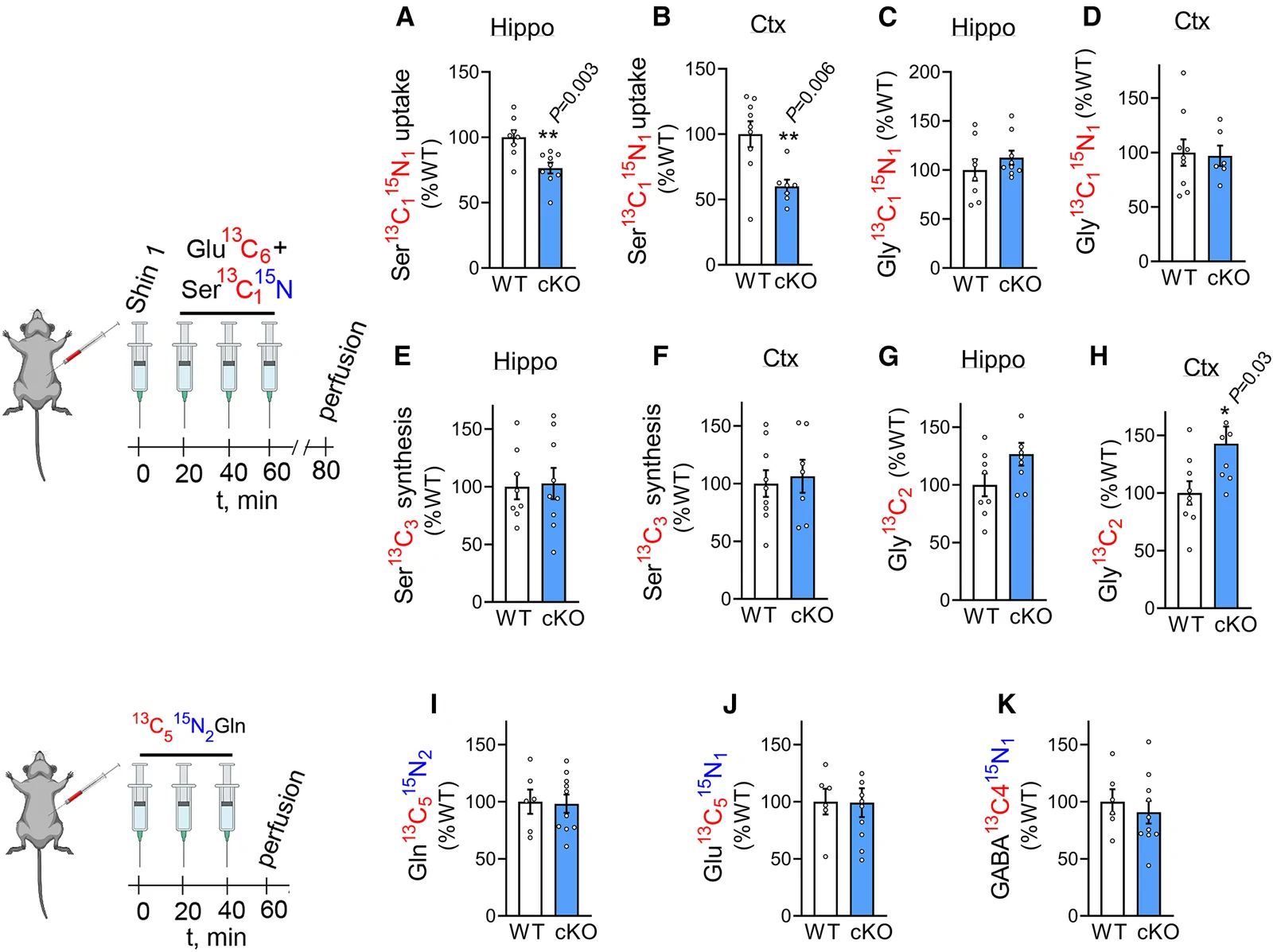
Isotope tracing reveals altered metabolism in *Slc38a2*-cKO mice (A and B) *Slc38a2*-cKO pups display lower uptake of Ser^13^C_1_^15^N in the hippocampus (A) and cerebral cortex (B). The data are mean ± SEM of 8–9 mice/group; Student’s *t* test. The values were normalized by the levels of Ser^13^C_1_^15^N in the serum. (C and D) *Slc38a2*-cKO pups display unaffected synthesis of Gly^13^C_1_^15^N from Ser^13^C_1_^15^N in the hippocampus (C) and cerebral cortex (D). The data are mean ± SEM of 8–9 mice/group; Student’s *t* test. (E and F) Synthesis of Ser^13^C_3_ from glucose ^13^C_6_ was unaffected in the hippocampi (E) and cerebral cortices (F) of *Slc38a2*-cKO pups. The data are mean ± SEM of 8–9 mice/group; Student’s *t* test. (G and H) Synthesis of Gly^13^C_2_ from Ser^13^C_3_ in the hippocampus (G) and cerebral cortex (H) of *Slc38a2*-cKO pups and WT littermates. The data are mean ± SEM of 8–9 mice/group; Student’s *t* test. (I) Uptake of glutamine^13^C_5_^15^N_2_ in the hippocampus of *Slc38a2*-cKO pups and WT littermates injected thrice with the tracer. The data are mean ± SEM of 6–10 mice/group; Student’s *t* test. (J) Synthesis of glutamate^13^C_5_^15^N_1_ from glutamine^13^C_5_^15^N_2_ in the hippocampus of *Slc38a2*-cKO pups and WT littermates. The data are mean ± SEM of 6–10 mice/group; Student’s *t* test. (K) Synthesis of GABA^13^C_5_^15^N_1_ from glutamine^13^C_5_^15^N_2_ in the hippocampus of *Slc38a2*-cKO pups and WT littermates. The data are mean ± SEM of 6–10 mice/group; Student’s *t* test. P11 pups were injected with 50 mg/kg Shin1 (intraperitoneal [i.p.]) followed by a mix of Ser^13^C_1_^15^N (50 mg/kg) and glucose^13^C_6_ (1 g/kg) every 20 min. Mice were transcardially perfused with ice-cold PBS, and tissues were collected for LC-MS analysis.

We found diminished hippocampal and cortical uptake of L-serine^13^C_1_^15^N_1_ in *Slc38a2*-cKO pups (Figures 4A and 4B). Synthesis of glycine^13^C_1_^15^N_1_ was unaffected in both regions (Figures 4C and 4D). The endogenous synthesis of serine^13^C_3_ from glucose^13^C_6_ was unaffected in the hippocampus and cortex of *Slc38a2*-cKO pups (Figures 4E and 4F). ^13^C-phosphoserine (m + 3) was detectable in the cerebral cortex and was unaffected in the *Slc38a2*-cKO mice (261,161 ± 44,612 and 173,474 ± 54,098 for wild-type [WT] and cKO, respectively, *p* = 0.23, 9 mice/group). This confirms the lack of compensatory change in the serine biosynthesis pathway. In the hippocampus, the glycine^13^C_2_ synthesis from glucose^13^C_6_ was unaltered (Figure 4G). However, in the cortex, there was an increase in the glycine^13^C_2_ synthesis from glucose^13^C_6_ (Figure 4H), suggesting an adaptive mechanism that maintains the brain glycine levels.

To verify if SLC38A2 carries out blood-to-brain influx of glutamine, we performed L-glutamine tracing *in vivo*. We found that the hippocampal uptake of glutamine^13^C_5_^15^N_2_ and the synthesis of glutamate^13^C_5_^15^N_1_ or GABA^13^C_4_^15^N_1_ were unaffected in *Slc38a2*-cKO pups, suggesting that SLC38A2 does not take up blood glutamine at this age.

We next asked whether brain serine deficiency in *Slc38a2*-cKO mice is sufficient to increase 1-deoxySL formation, as observed following -SG feeding (Figure 1F). Sphingolipidomic analysis in the hippocampus revealed only a modest 20% increase in a single deoxydihydroceramide species, doxCer (m18:0/18:0) (Figure S5A). While the -SG diet increased the Ala/Ser ratio by 90% (Figure 1E), the *Slc38a2*-cKO had only a 35% increase in the Ala/Ser ratio (Figure S5B). These results suggest that, unlike dietary restriction, the increase in hippocampal Ala/Ser ratio by *Slc38a2* deletion is relatively mild and remains below the threshold required to drive substantial 1-deoxySL accumulation.

### Slc38a2-cKO pups display defects in excitatory synapse ultrastructure but normal motor behavior

**Figure 5:**
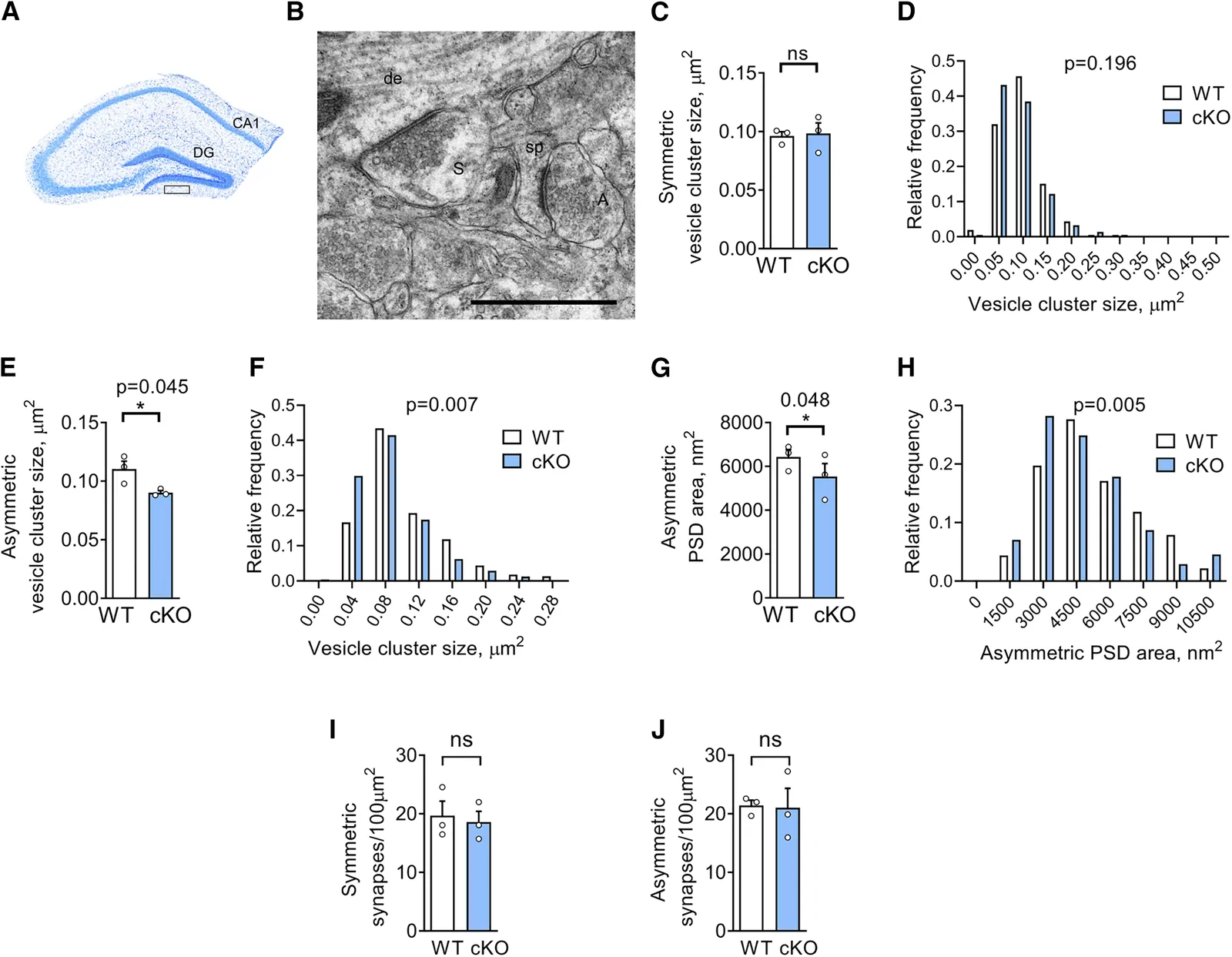
Abnormal excitatory synapses in *Slc38a2*-cKO pups (A) Diagram of the molecular layer of the medial blade of the dentate gyrus (DG) examined for synaptic morphology. (B) Representative electron micrograph of a symmetric and asymmetric synapse in P14 *Slc38a2*-cKO pups. A, asymmetric synapse; de, dendrite; S, symmetric synapse; sp, dendritic spine. Scale bars, 1 μm. (C and D) Symmetric synapses of P14 *Slc38a2*-cKO pups exhibit a normal presynaptic area (C) and size distribution (D). Data are from 213 (WT) and 234 (cKO) synapses. Student’s *t* test and Kolmogorov-Smirnov test for frequency distribution. (E and F) Asymmetric synapses of P14 *Slc38a2*-cKO pups exhibit a smaller vesicle cluster and skewed distribution toward lower values. Data are from 215 (WT) and 225 (cKO) synapses. Student’s *t* test and Kolmogorov-Smirnov test for frequency distribution. (G and H) P14 *Slc38a2*-cKO pups exhibit a smaller PSD area (G), and the distribution was skewed toward lower values (H). Data are from 215 (WT) and 225 (cKO) synapses. Student’s *t* test and Kolmogorov-Smirnov test for frequency distribution. (I and J) The number of symmetric (I) and asymmetric (J) synapses was unaltered in P14 *Slc38a2*-cKO pups. The data are mean ± SEM of 3 mice/group; Student’s *t* test.

Because both L-serine and D-serine are important for synaptic formation,^32^^,^^33^^,^^34^ we investigated the effect of *Slc38a2*-cKO on the ultrastructure of synapses in the molecular layer of the medial blade of the dentate gyrus (Figure 5A), a region that exhibits intense synaptogenesis at this stage.^35^ Synapses were classified morphologically as symmetric (predominantly GABAergic) or asymmetric (predominantly glutamatergic) (Figure 5B). Symmetric synapses were similar in size in *Slc38a2*-cKO pups and WT littermates (Figures 5C and 5D). In contrast, asymmetric synapses were selectively affected in *Slc38a2*-cKO pups, exhibiting a 25% reduction in synaptic vesicle cluster size (Figure 5E), with the overall distribution shifted toward smaller values (Figure 5F). The postsynaptic density (PSD) area of asymmetric synapses was also reduced relative to WT littermates (Figure 5G), with a corresponding leftward shift in the size distribution (Figure 5H). The density of both symmetric and asymmetric synapses, however, was unchanged (Figures 5I and 5J).

We did not detect changes in the expression of the neuronal markers NeuN and PSD-95 in *Slc38a2*-cKO mice (Figure S6A). Similarly, expression of the astrocytic marker GFAP was unchanged. None of the metabolic enzymes for L-serine, D-serine, and glycine were affected in *Slc38a2*-cKO pups as well. Likewise, bulk RNA-seq showed changes in only a handful of genes, suggesting that Slc38a2-cKO does not lead to substantial changes in gene expression (Table S5).

We also assessed developmental motor behavior in *Slc38a2*-cKO pups and control littermates. *Slc38a2* deletion had no detectable effect on negative geotaxis, grip strength, or forelimb and hindlimb suspension tests, suggesting that the pups are behaviorally resilient to the metabolic and synaptic alterations caused by loss of *Slc38a2* (Figure S7).

### Slc38a2 deletion under a serine-restricted diet

**Figure 6:**
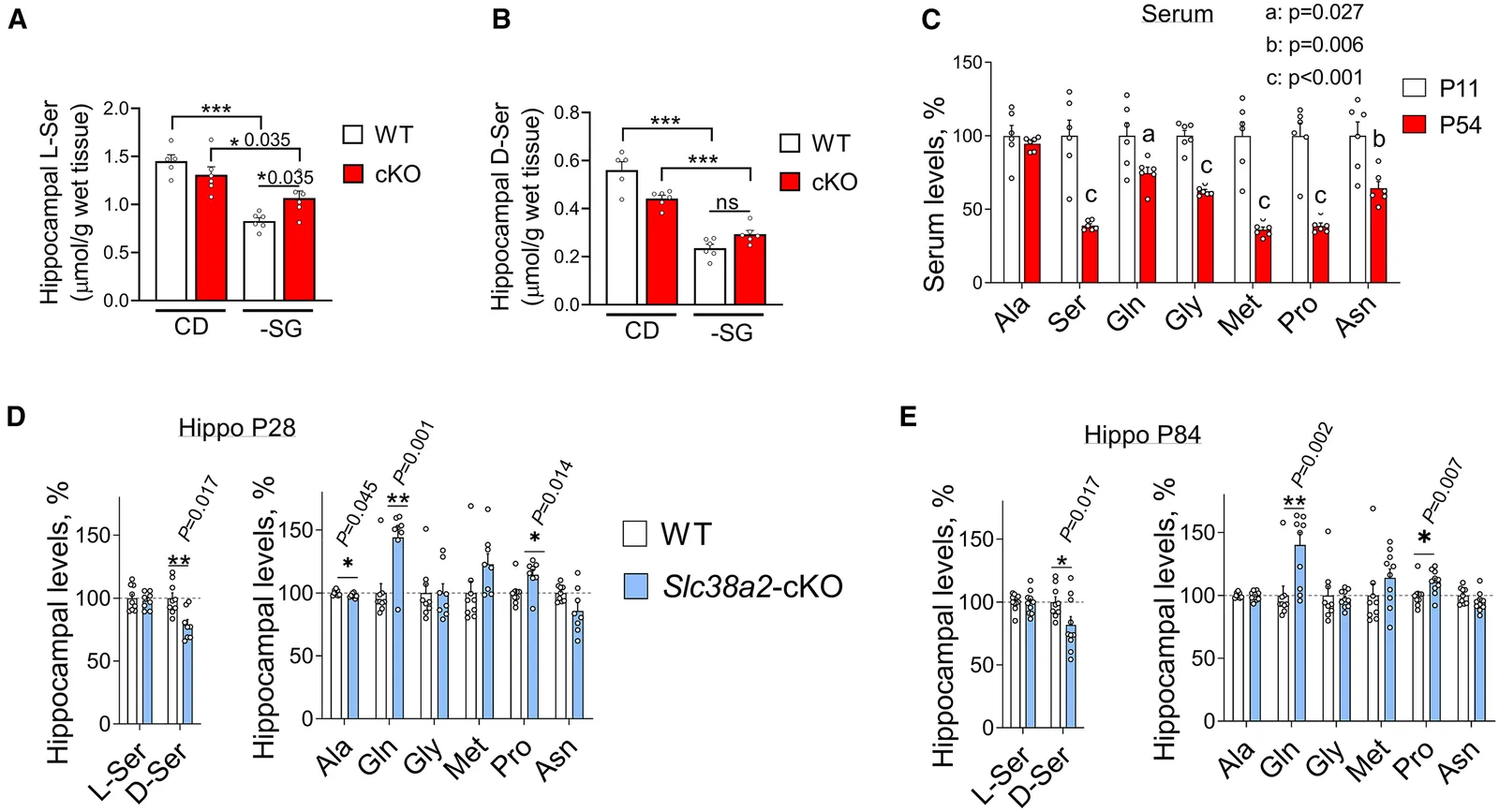
Slc38a2 deletion under a serine-restricted diet and use of other substrates by the transporter in older mice (A) Four-week-old *Slc38a2*-cKO and WT littermates were fed with either -SG or CD diet for 10 days and hippocampal L-serine levels were determined by HPLC (*n* = 5–6 mice/group). Statistics: one-way ANOVA with Holm-Sidak’s multiple comparison test. ∗∗∗*p* < 0.001. (B) Same as (A) but with hippocampal D-serine levels (*n* = 5–6 mice/group). Statistics: one-way ANOVA with Holm-Sidak’s multiple comparison test. ∗∗∗*p* < 0.001. (C) Serum levels of SLC38A2 *in vitro* substrates were lower in P54 than in P11 WT mice. The data are mean ± SEM of 6 mice/group; Student’s *t* test. (D) Lower levels of D-serine in the hippocampus of P28 *Slc38a2*-cKO mice monitored by HPLC (left graph). Absolute values of L-serine are (in μmol/g wet tissue, average ± SEM) 1.78 ± 0.06 (WT) and 1.75 ± 0.04 (Slc38a2-cKO). Absolute values of D-serine are 0.58 ± 0.02 (WT) and 0.46 ± 0.02 (Slc38a2-cKO). Hippocampal levels of additional SLC38A2 substrates in P28 mice monitored by LC-MS show higher glutamine and proline concentrations compared to WT littermates (right graph). The data are mean ± SEM of 8–9 mice/group; Student’s *t* test. (E) Lower levels of D-serine in the hippocampus of P84 *Slc38a2*-cKO mice monitored by HPLC (left graph). Absolute values of L-serine are (in μmol/g wet tissue, average ±SEM) 1.41 ± 0.03 (WT) and 1.41 ± 0.04 (Slc38a2-cKO). Absolute values of D-serine are 0.51 ± 0.01 (WT) and 0.42 ± 0.03 (Slc38a2-cKO). Hippocampal levels of additional SLC38A2 substrates in P84 mice monitored by LC-MS show higher glutamine and proline concentrations compared to WT littermates (right graph). The data are mean ± SEM of 8–9 mice/group; Student’s *t* test. The data are mean ± SEM of 8–9 mice/group; Student’s *t* test.

Four-week-old *Slc38a2*-cKO mice were fed the -SG diet, and levels of L-serine and D-serine were measured by HPLC in the hippocampus (Figures 6A and 6B). We observed no additive effect of the -SG diet and *Slc38a2* deletion, suggesting that both perturb the same biochemical pathway. Unexpectedly, L-serine levels in -SG-fed *Slc38a2*-cKO mice were even higher than those in diet-matched WT controls (Figure 6A). This finding raises the possibility that, under conditions of reduced circulating L-serine, SLC38A2 may contribute to the equilibration or efflux of L-serine from the brain, consistent with its localization to both luminal and abluminal endothelial membranes.

### Brain levels of additional SLC38A2 substrates in older mice

While luminal SLC38A2 is well positioned to mediate L-serine uptake, abluminal SLC38A2 may instead promote amino acid efflux from the brain. The direction of net amino acid flux across the BBB is expected to depend, at least in part, on their circulating concentrations. We found that, among the *in vitro* substrates of SLC38A2, all except alanine are significantly lower in the serum of 8-week-old mice (P54) relative to P11 pups, possibly reflecting the dietary transition from a milk-based diet to standard chow (Figure 6C). Since D-serine is only a small fraction of total serine (4%),^36^ total serum serine measured by LC-MS almost entirely reflects L-serine. These lower circulating concentrations of amino acids, including L-serine, would be expected to reduce L-serine influx into the brain while favoring equilibrative efflux. We therefore expanded our investigation to older mice, in which circulating serum serine levels are lower, and brain glutamine production by glutamine synthetase is several-fold higher than in pups.^37^

Strikingly, LC-MS indicates that glutamine largely accumulated in the hippocampus of both juvenile and adult *Slc38a2*-cKO mice (Figures 6D and 6E; Tables S6), suggesting that glutamine is a physiological *in vivo* substrate of SLC38A2 and is normally cleared from the brain via abluminal SLC38A2 at these ages. Proline also accumulated in the brains of older *Slc38a2*-cKO mice, consistent with a similar mode of transport by SLC38A2. In older *Slc38a2*-cKO mice, L-serine levels were preserved, but hippocampal D-serine was still reduced as measured by HPLC (Figures 6D and 6E). The persistent effect on D-serine levels suggests that despite lower circulating serine levels, older mice can still import serine via luminal SLC38A2.

## Discussion

SLC38A2 was not previously thought to be present at the luminal endothelial membrane or to contribute to serine metabolism. Using a combination of metabolomics, isotope tracing, and physiological experiments, we established SLC38A2 as a bona fide L-serine transporter at the BBB. The biochemical changes observed in -SG-fed animals, and to a smaller extent in *Slc38a2-*cKO mice, indicate that blood-to-brain L-serine influx makes a major contribution to brain D-serine synthesis. D-serine is synthesized in the brain by the enzyme serine racemase^38^ and serves as a physiological co-agonist of NMDARs, thereby regulating multiple NMDAR-dependent processes.^25^^,^^39^^,^^40^ These include synaptic plasticity,^41^^,^^42^^,^^43^^,^^44^ activity-dependent dendritic spine growth,^33^ and neurotoxicity.^45^ Interestingly, serine racemase is highly expressed in the forebrain areas, especially in dendritic areas,^46^ where it overlaps with SLC38A2 expression, which is also enriched in somatodendritic areas in the forebrain.^20^ SLC38A2 expression is reduced in the dorsolateral prefrontal cortex of schizophrenic subjects,^47^ suggesting it may also change D-serine metabolism and contribute to NMDAR hypofunction in the disease.

SLC38A2 has a broad range of substrates, though L-serine seems to be particularly effective.^13^^,^^27^ One possible explanation for the quite selective decrease in L-serine we observed in Slc38a2-cKO brains is the high demand for L-serine by the developing brain.^3^ In this context, any decrease in brain influx will substantially affect the steady-state levels of L-serine. Other SLC38A2 substrates may be less affected due to lower demand or compensatory transport by other SLCs.

D-serine is present in the circulation at very low concentrations (∼1 μM) and crosses the BBB poorly, indicating that the vast majority of brain D-serine is synthesized locally within the CNS.^48^ SLC38A2 is also unlikely to mediate significant D-serine transport, as it transports L-serine with ∼22-fold greater efficiency than D-serine, based on the Vmax/Km ratio.^27^ Moreover, serine racemase exhibits low affinity for L-serine (Km ∼10 mM),^38^ suggesting that reductions in brain L-serine availability would be expected to directly constrain D-serine synthesis. Within this framework, the lower brain D-serine levels observed in *Slc38a2*-cKO pups are most likely attributable to reduced L-serine substrate availability rather than to a direct defect in D-serine transport across the BBB.

**Figure 7:**
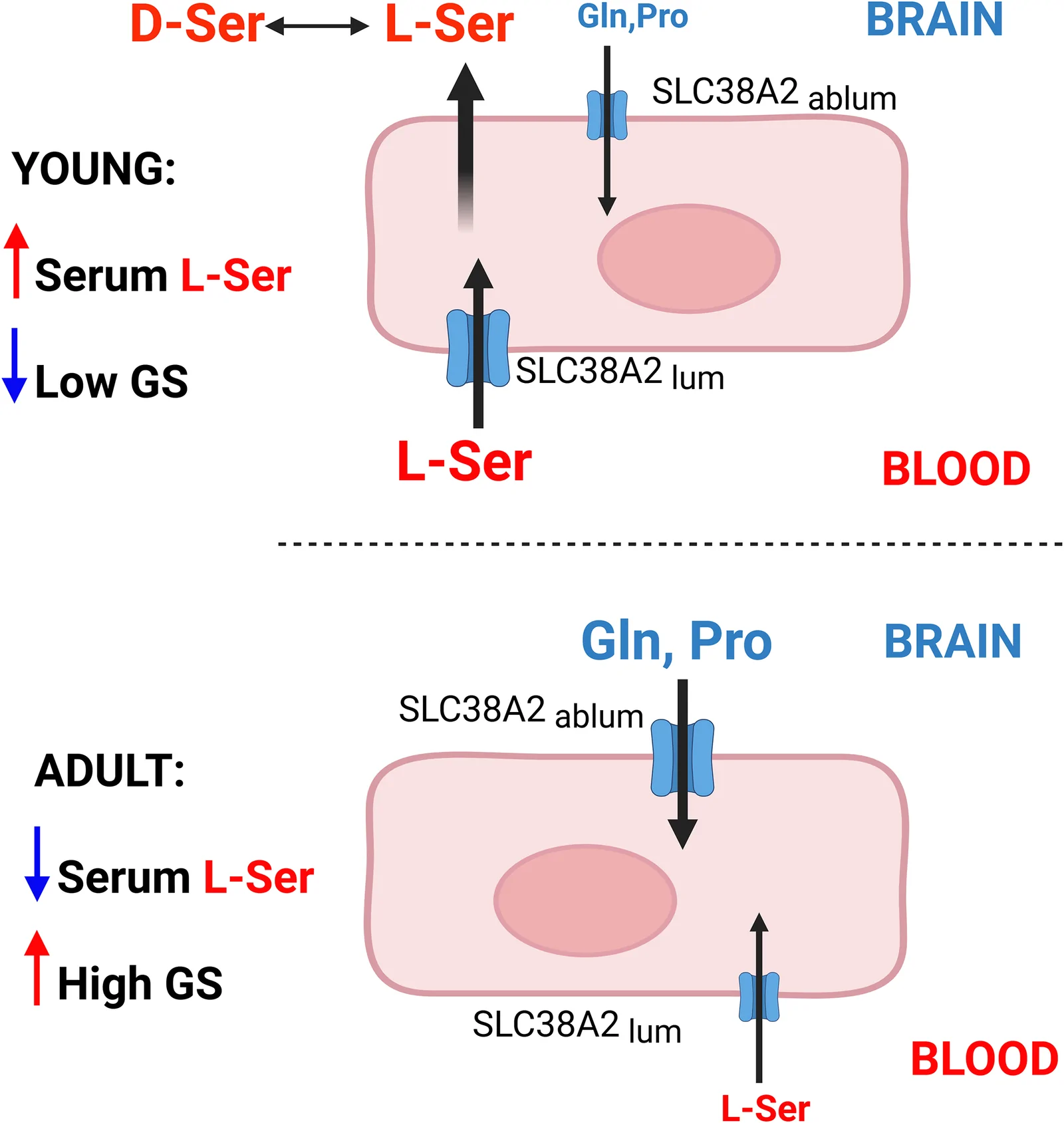
Schematic of the proposed developmental role of SLC38A2 at the luminal and abluminal membranes In young preweaning mice, elevated serum L-serine levels favor its uptake via luminal SLC38A2, thereby facilitating L-serine delivery for D-serine synthesis (upper image). In contrast, abluminal SLC38A2 is predicted to be less active owing to an unfavorable concentration gradient, characterized by higher serum glutamine and proline levels and lower brain glutamine synthetase (GS).^37^ In older mice, lower serum levels of L-serine and other SLC38A2 substrates are expected to limit luminal transport. Under these conditions, we infer that an abluminal transport could be favored by a steeper brain-to-blood substrate gradient and by maximal brain GS expression.

The molecular mechanisms affecting SLC38A2 expression patterns, post-translational regulation, or the expression of compensatory transporters during development are unknown. However, there are developmental changes in SLC38A2 substrate concentration gradients across the BBB when comparing P11 to adult mice. There is almost a 3-fold decrease in serum L-serine in adult mice compared to P11 (Figure 4). This aligns with the higher influx of L-serine via the luminal SLC38A2 species in young mice, which may be facilitated by the higher concentration gradient (Figure 7). On the other hand, there is a several-fold increase in endogenous brain glutamine production through the glutamine synthetase from P11 to adults.^2^^,^^37^ Older mice and humans export excess glutamine produced in the brain into the blood via unknown transporters.^49^^,^^50^ Therefore, the accumulation of glutamine in juvenile and older *Slc38a2-cKO* mice implies a role of SLC38A2 in L-glutamine export, which is facilitated by the higher production of brain glutamine in older ages (Figure 7). In juvenile and adult mice, we observed accumulation of proline in the brains of Slc38a2-cKO mice. Proline can be derived from ornithine and is involved in ammonia disposal through its conversion to glutamate.^51^ Likewise, glutamine incorporates ammonia during its biosynthesis. We propose that an SLC38A2-mediated efflux of glutamine and proline from the brains of older animals contributes to the removal of excess ammonia, which is toxic at elevated concentrations.^52^ Accordingly, both glutamine and proline were previously shown to be released from the brain.^50^^,^^53^

It is noteworthy that older *Slc38a2*-cKO mice also exhibit lower brain D-serine levels, indicating that L-serine import via luminal SLC38A2 remains active in older mice. By comparison, the expression of other serine transporters at the BBB is too low in adults to substantially regulate adult brain metabolism.^1^^,^^3^ This positions SLC38A2 as the stronger candidate for mediating the uptake of dietary L-serine in adults. Indeed, deletion of *Slc38a2* was not additive to dietary L-serine restriction with respect to brain L-serine and D-serine levels, suggesting that they act within the same pathway.

Tracing experiments with serine (^13^C_1_ and ^15^N_1_) and glucose (^13^C_6_) showed that synthesis of glycine was unaffected in the hippocampus but was higher in the cortex of *Slc38a2*-cKO pups. This likely reflects more efficient serine/glycine interconversion via SHMT1 or SHMT2 to compensate for reduced L-serine uptake into the brain. Whether this effect is mediated primarily by the cytosolic or mitochondrial SHMT isoform remains to be determined.

SLC38A2 is expressed in the peripheral vasculature, and its deletion has been reported to lower blood pressure.^54^ For technical reasons, blood pressure could not be measured in pups. Nevertheless, *Slc38a2*-cKO pups showed no detectable metabolic changes in serum, supporting the interpretation that the observed phenotype is predominantly brain-specific.

The most prominent phenotypic change in *Slc38a2*-cKO mice is the reduction in synaptic vesicle cluster size and PSD area at excitatory synapses. This phenotype resembles the synaptic abnormalities previously observed following deletion of other endothelial serine transporters, including SLC38A5 and SLC1A4.^1^^,^^3^ However, the synaptic changes in Slc38a2-cKO were milder and likely compensated by other mechanisms, as the motor behavior of Slc38a2-cKO was preserved. In those studies, transporter deletion in pups also decreased brain levels of additional transporter substrates, such as threonine, indicating that the transporters are not selective for L-serine. *Slc38a2* deletion promoted gentler phenotypes than the -SG diet, particularly in deoxysphingolipid synthesis and gene expression. This suggests the presence of additional L-serine transporters at the BBB that may partially compensate for *Slc38a2* deletion. Altogether, our results highlight the importance of dietary L-serine for brain metabolism and synapse formation.

Inhibitors of L-serine transport through luminal SLC38A2 could reduce D-serine synthesis and may therefore be beneficial in conditions associated with L-serine overproduction, astrogliosis, and NMDAR overactivation, including epilepsy^55^^,^^56^ and traumatic brain injury.^44^^,^^57^ Endothelial SLC38A2 may also represent a targetable metabolic vulnerability in brain tumors. Given that glioblastoma multiforme (GBM) growth relies on the transport of dietary L-serine across the BBB^58^ and that SLC38A2 is overexpressed in GBM,^59^ SLC38A2 inhibitors could limit tumor growth by restricting access to this essential metabolite.

### Limitations of the study

The magnitude of L-serine and other metabolic changes in the *Slc38a2*-cKO mice was lower than in SG-fed mice, suggesting that SLC38A2 is a contributor to brain L-serine transport, but not necessarily the major transporter. Alternatively, *Slc38a2* deletion may induce compensatory mechanisms, such as activation of other transporters that are not engaged by dietary serine/glycine restriction. Future experiments combining *Slc38a2* deletion with other endothelial serine transporters may clarify this issue. Furthermore, -SG-fed animals display changes in transcription, and the effects were also more pronounced than in *Slc38a2*-cKO mice. Future validation of selected targets at the protein level could help determine the biological significance of the observed transcriptomic alterations, given that changes in transcript abundance do not necessarily translate into corresponding changes in protein expression. The accumulation of glutamine and proline in the brains of older *Slc38a2*-cKO mice is compatible with a decrease in the export of these amino acids from the brain. However, the possible export of glutamine and other substrates by the abluminal SLC38A2 has not been directly measured. Experiments employing selective inactivation of the luminal vs. abluminal SLC38A2 species would be required to elucidate their roles with different substrates. Finally, although both sexes were included in the experiments, the influence of sex was not investigated in this study.

## Resource availability

### Lead contact

Further information and requests for resources and reagents should be directed to and will be fulfilled by the lead contact, Herman Wolosker, hwoloske@technion.ac.il

### Materials availability

There are restrictions on the availability of Slc38a2 fl/fl mice due to the lack of a centralized external repository for their distribution and our need to maintain the stock. We are glad to share Slc38a2 fl/fl mice with reasonable compensation by the requestor for processing and shipping.

### Data and code availability

• The RNA-seq data generated in this study are available from GEO, accession number GSE330116. Metabolomics spectra are publicly available in the massIVE database: https://massive.ucsd.edu/ (dataset MSV000101871). Sphingolipidomics spectra are available in the massIVE database (dataset MSV000101922).
• This study did not generate new code.
• Any additional information required to reanalyze the data reported in this paper is available from the lead contact upon request.

## Acknowledgments

We thank Dr. LiHi Shaulov for expert electron microscopy, and Ari Feder for the help in metabolomics analysis. This work was supported by the Laura and Isaac Perlmutter Metabolomics Center, the 10.13039/501100003977Israel Science Foundation/10.13039/501100001711Swiss National Science Foundation grant no. 1698/25 to S.S., T.H., and H.W., the Israel Science Foundation grant no. 279/24, the Allen and Jewel Prince Center for Neurodegenerative Diseases, and the Rappaport Family Institute for Research in the Medical Sciences to H.W.

## Author contributions

A.K.T., A.L., and C.S. performed the experiments; A.M. and S.B. performed sphingolipidomics; I.A. and B.A. performed metabolomics; Y.H., S.S., F.A.C., T.H., and I.R. analyzed the data; H.W. analyzed the data and wrote the paper.

## Declaration of interests

The authors declare no conflict of interests.

## Declaration of generative AI and AI-assisted technologies in the writing process

During the preparation of this work, the authors used ChatGPT in order to improve the prose. After using this tool or service, the authors reviewed and edited the content as needed and take full responsibility for the content of the publication.

## STAR★Methods

### Key resources table

**Table:** 

REAGENT or RESOURCE	SOURCE	IDENTIFIER
**Antibodies**		

Rabbit polyclonal anti-Slc38a2	Ref.^20^	N/A
Mouse monoclonal anti-actin	MP Biomedicals	RRID:AB_2335127
Mouse anti-GFAP	Calbiochem	RRID:AB_2294571
Rabbit polyclonal NeuN	Cell Signaling Technology	RRID:AB_2651140
Rabbit polyclonal anti-Phgdh	Cell Signaling Technology	RRID:AB_2750870
Rabbit polyclonal anti SHMT1	Cell Signaling Technology	RRID:AB_2799957
Rabbit polyclonal anti SHMT2	Cell Signaling Technology	RRID:AB_2798018
Rabbit polyclonal anti-PSAT1	Novus Biologicals	RRID:AB_2172600
Mouse monoclonal anti-Serine Racemase (SR)	Santa Cruz Biotechnology	RRID:AB_10847683
Anti-gapdh	Sigma-Aldrich	RRID:AB_796208
Mouse monoclonal anti PSD-95	StressMarq Biosciences	RRID:AB_2698081
Goat polyclonal anti-CD31	R&D systems	RRID:AB_2161028

**Chemicals, peptides, and recombinant proteins**		

D-glucose (U-^13^C_6_)	Cambridge Isotope Laboratories	Cat CLM-1396-PK
L-serine (^13^C_1_,^15^N)	Cambridge Isotope Laboratories	Cat CNLM-7814-PK
L-Glutamine (^13^C_5_,^15^N_2_)	Cambridge Isotope Laboratories	Cat CNLM-1275-H
Glutamine, L-[^14^C(U)]	Perkin Elmer	Cat NEC451050UC, lot 2700604
Methyl-D-glucose, 3-*O*-[Methyl-^3^H(N)]	Perkin Elmer	Cat NET379001MC, lot 657433
Serine, L-[^14^C]	Perkin Elmer	NEC827050UC
Blotto, non-fat dry milk	Santa Cruz Biotechnology	Cat sc-2325
Bovine Serum Albumin	Sigma Aldrich	Cat A8022
cOmplete™, Mini Protease Inhibitor Cocktail	Roche	Cat 11836153001
PhosSTOP, phosphatase inhibitor tablets	Roche	Cat 04 906 837 001
Fluorescein	Sigma Aldrich	Cat F6377
Heparin	Sigma Aldrich	Cat H3149
Paraformaldehyde	Merck	Cat 8.18715.1000
0-phtalodialdehyde	Sigma Aldrich	Cat P1378
Boc-L-Cysteine	Sigma Aldrich	Cat 15411
Screw cap micro tube, 2 mL, PP	Sarstedt	Cat 72.694.005
Zirconium Oxide Beads, 1 mm	Next Advance	Cat ZrOB10
Ultima Gold XR	Perkin Elmer	Cat 6013119
Glutaraldehyde, 25% solution	Electron Microscopy Sciences	Cat 16220
Uranyl acetate	Electron Microscopy Sciences	Cat 22400–4

**Critical commercial assays**		

Protein Assay Dye Reagent	Bio-Rad	Cat 5000006
SuperSignal West Dura substrate kit	Thermo Scientific	Cat 34075

**Deposited data**		

RNAseq	Gene Expression Omnibus https://www.ncbi.nlm.nih.gov/geo/	access GSE330116, reviewer token: mfmfaaimjlwzdqn.
Metabolomics	massIVE database https://massive.ucsd.edu/	dataset MSV000101871
Sphingolipidomics	massIVE database https://massive.ucsd.edu/	dataset MSV000101922

**Experimental models: Organisms/strains**		

Slc38a2 fl/fl	This study	N/A
B6.Cg-Tg(Tek-cre)1Ywa/J (Tie2-Cre)	Jackson Laboratory	Strain #:008863

**Oligonucleotides**		

Slc38a2_181259_F, 5′-CAGTGTCACCCCAGAACCAG-3′	Sigma	N/A
slc38a2_181259_R, 5′-AAGGAAGGCAGGTAGAGGTGG-3′	Sigma	N/A
oIMR1084, 5′GCGGTCTGGCAGTAAAAACTATC 3′	Sigma	N/A
oIMR1085, 5′GTGAAACAGCAT TGCTGTCACTT 3′	Sigma	N/A

**Software and algorithms**		

Skyline	MacCoss Lab Software	Version 22.2.0.527
GraphPad Prism	GraphPad Software	Version 8.2.1.
MetaboAnalyst 6.0	N/A	https://www.metaboanalyst.ca/home.xhtml

### Experimental model and study participant details

The *Slc38a2*-cKO mice were generated from the targeted allele *Slc38a2*^tm1a(KOMP)Wtsi^ obtained from the Wellcome Trust Sanger Institute on a C57BL/6N genetic background. The knockout-first allele was converted to the floxed tm1c allele by Flp recombinase-mediated excision of the selection cassette. To generate endothelial-specific conditional knockout mice, *Slc38a2*^fl/fl^ mice were crossed with Tie2Cre transgenic mice (Strain #: 008863, The Jackson Laboratory). The breeding scheme consisted of crossing females *Slc38a2*^fl/fl^ with Tie2Cre^fl/+^ or Tie2Cre^fl/fl^ males. Mice were housed in a temperature-controlled facility under pathogen-free conditions in a 12-hour light/dark cycle with unrestricted access to food (Altromin 1324 chow diet) and water. All animal experiments were approved by the Committee for the Supervision of Animal Experiments at the Technion – Israel Institute of Technology and were conducted in accordance with institutional and national ethical guidelines (Ethical approval IL-026-02-22, IL-009-01-26, IL-037-03-22, IL-010-01-26). In all experiments, mice of both sexes were used. Sex was not balanced across groups and was not independently considered in the analysis.

### Method details

#### Genotyping

Genomic DNA was isolated from mouse tail biopsies collected for routine genotyping. Tail tips were lysed using DirectPCR Lysis Reagent (Viagen Biotech) according to the manufacturer’s instructions. PCR amplification was carried out in a thermal cycler using the following program: 95°C for 2 min followed by 36 cycles at 95°C for 40 s, 64°C for 30 s, and extension at 72°C for 30s. The following primer pairs were used: Slc38a2_181259_Fand slc38a2_181259_R for the floxed allele (593 bp fragment) and oIMR1084 and oIMR1085 for Tie2-Cre (100 bp fragment).

#### Serine/glycine dietary restriction

Three- or four-week-old mice were fed for 10 days with a control amino acid diet containing defined amounts of individual amino acids (CD) (Inotiv, TD. 01084) or a serine and glycine-restricted diet (-SG) in which L-serine was decreased from 3.5g/Kg to 0.35g/Kg and glycine from 23.3 g/Kg to 1 g/Kg.

#### HPLC analysis

Brain samples were weighed and placed into 2 ml screw cap homogenization tubes prefilled with 1.0 mm ceramic beads and 1:15 wet weight:volume cold extraction solution (-20°C) consisting of methanol:acetonitrile:water (5:3:2, v/v/v). The samples were homogenized using a Precellys 24 tissue homogenizer (Bertin Technologies) at 4°C (4 cycles × 30 s at 6000 rpm, with a 30 s gap between cycles) and centrifuged at 18,000 × g for 15 min at 4°C. The supernatant was derivatized with o-phtalodialdehyde and Boc-L-cysteine and separation of L- and D-enantiomers by HPLC was carried out as previously described.^43^

#### Metabolomic analysis

For metabolite profiling, brain, liver, or kidney samples were homogenized as described above for HPLC analysis. For serum metabolomic analysis, 10 μl of serum was transferred into a microtube containing 90 μl of cold (-20°C) ethanol:acetonitrile (3:1, v/v). The samples were vortexed at 4°C for 10 min and centrifuged at 18,000 × g for 15 min at 4°C. The supernatants were transferred into HPLC glass vials. All samples were stored at −80°C until LC-MS analysis.

LC-MS analysis was performed as previously described.^60^ Briefly, a Thermo Vanquish Flex ultra-high-performance liquid chromatography (UHPLC) system coupled to an Orbitrap Exploris 240 Mass Spectrometer (Thermo Fisher Scientific) was used at a resolution of 120,000 at 200 m/z, electrospray ionization, and polarity switching mode to detect both positive and negative ions across a mass range of 67–1000 m/z. Five microliters of samples were injected into a ZIC-pHILIC column (SeQuant; 150 mm × 2.1 mm, 5 μm; Merck), and compounds were separated using a 15-min mobile phase gradient, starting at 20% aqueous mobile phase of 20 mM (NH_4_)_2_CO_3_, adjusted to pH 9.2 with 0.1% NH_4_OH (25%), plus 80% acetonitrile, ending at 20% acetonitrile.

All metabolites were detected with mass accuracy below 5 ppm. Data acquisition was performed using Thermo Xcalibur 4.4. Peak areas of different metabolites were determined using Skyline 24.1.0.414 software. Metabolites were identified by matching the exact mass of the singly charged ion and the retention time to a corresponding standard, using an in-house library compiled from running commercial standards for all detected metabolites. Natural abundance values were corrected in each isotope tracing experiment.

#### Sphingolipidomics

Extraction was performed with MMC buffer (methanol: methyl tert-butyl ether: chloroform, 4:3:3, v/v/v) with added internal standards (see below) by agitation in a Thermomixer (Eppendorf) for 60 min at 37°C. After centrifugation (16 100 rpm, 10 minutes, 37°C), the single-phase supernatant was collected, dried under N2, and stored at -20°C. Before analysis, lipids were dissolved in 50μL of MeOH Thermomixer (Eppendorf), 60 minutes, 37°C) and separated on a C18 ACQUITY UPLC CSH (Waters, 150mm x 2.1 mm x 1.7μm) using gradient elution with A) Acetonitrile: Water (6:4) with 10mM ammonium acetate and 0.1% formic acid and B) Isopropanol: Acetonitrile (9:1) with 10mM ammonium acetate and 0.1% formic acid at a flow rate of 260μL/minute using Transcend UHPLC pump (Thermo Fisher Scientific) at 50°C. The following gradient was used: 0min 30% (B), 0.5min 30% (B), 2min 43% (B), 3.3min 55% (B), 12min 75% (B), 18min 100% (B), 25min 100% (B), 25.5min 30% (B), 29.5min 30% (B). Eluted lipids were analyzed on a Q-Exactive MS (Thermo Fisher Scientific) in positive mode using heated electrospray ionization (HESI, Sheath gas flow rate = 40, Aux gas flow rate = 10, Sweep gas flow rate = 0, spray voltage = 3.50, capillary temperature = 320°C, S-lens RF level = 50.0, Aux gas heater temperature = 325°C). MS2 fragmentation spectra were recorded in data-dependent acquisition mode with a top10 approach and constant collision energy of 25 eV. 140 000 resolution was used for full MS1 and 17 500 for MS2. Peak integration was performed with Skyline daily and Compound Discoverer 3.3 (Thermo Fisher Scientific). Lipids were identified by predicted mass (resolution 5 ppm), retention time (RT), and specific fragmentation patterns (mzVault), using in-house-made Lipidcreator and MSDIAL (MSDIAL-TandemMassSpectralAtlas-VS69-Pos) compound databases. Next, lipid concentrations were normalized to the internal standards (one per class) and to the wet weight (pmol/mg).

Added internal standards: d5-1-deoxymethylsphinganine (m17:0, 860476, Avanti Polar Lipids) 100pmol/sample, 1-deoxyDHCeramide (m18:0/12:0, 860460P, Avanti Polar Lipids) 100pmol/sample, 1-deoxyCeramide (m18:1/12:0, 860455, Avanti Polar Lipids) 100pmol/sample, DHCeramide (d18:0/12:0, 860635 Avanti Polar Lipids) 100pmol/sample, ceramide (d18:1/12:0, 860512, Avanti Polar Lipids) 100pmol/sample, sphingomyelin (d18:1/12:0, 860583, Avanti Polar Lipids) 100pmol/sample, glucosylceramide (d18:1/8:0, 860540, Avanti Polar Lipids) 100pmol/sample.

#### RNAseq

Total RNA was extracted from hippocampi of either 3-week old mice fed with -SG or CD diet for 10 days (*n* = 5 mice/group) or P11 mice *Slc38a2*-cKO and WT littermates (*n* = 5/group) using RNeasy Mini Kit (Qiagen, cat 74106). Three hundred and fifty μL of RLT buffer was added to each sample, and they were homogenized using the Polytron PT3000 Homogenizer (Kinematica). After homogenization, the samples were centrifuged for 3 min at 13000 × g, and the supernatant was transferred to a new tube for automated extraction using the QIAcube Connect (Qiagen). The RINe value for all samples ranged from 9.7 to 9.9, indicating very high quality.

RNA-seq libraries were prepared simultaneously using the NEBNext Ultra II Directional RNA Library Prep Kit for Illumina (NEB, cat E7760), according to the manufacturer’s instructions. Two hundred and fifty ng of total RNA served as the starting material. mRNA pull-down was performed using the NEBNext® Poly(A) mRNA Magnetic Isolation Module (NEB, cat E7490). Library quality control was conducted by measuring library concentration with Qubit (Invitrogen) using the Equalbit dsDNA HS Assay Kit (Vazyme, cat EQ121) and assessing size distribution with the TapeStation 4200 (Agilent) using the High Sensitivity D1000 kit (Agilent, cat 5067-5584). All libraries were pooled into a single tube at equal molarity. RNA-seq data were generated on the Illumina NextSeq 2000, using P2 100 cycles (Read 1–100; Index 1–8; Index 2–8; Read 2–0) (Illumina, cat 20046811).

Quality control was assessed with Fastqc (v0.12.1). Reads were trimmed for adapters, with a minimum Phred quality score of 20 and a minimum length of 35, using CUTADAPT (v4.4). Single reads (100 bp) were aligned to Mus musculus (GRCm39) reference genome (https://ftp.ensembl.org/pub/release-111/fasta/mus_musculus/dna/Mus_musculus.GRCm39.dna.primary_assembly.fa.gz) and annotation file (https://ftp.ensembl.org/pub/release-111/gtf/mus_musculus/Mus_musculus.GRCm39.111.gtf.gz) using STAR (v2.7.10b) with a mismatch ratio of less than 0.2, and the minimum and maximum intron sizes were set to 21 and 1,000,000, respectively. The number of reads per gene was counted using STAR “--quantMode GeneCounts” (v2.7.10b) in ‘reverse’ mode. Normalization and differential expression analyses were performed using the DESeq2 R package (v1.41.8). Two factors determine the threshold for significantly differentially expressed genes: an adjusted *p*-value of ≤ 0.05 and the ‘base-mean independent filtering’ threshold, which is calculated by the DESeq2 algorithm for each comparison. GO enrichment analysis was determined using ShinyGO 0.85. The *p*-values were corrected for multiple testing using the Benjamini-Hochberg FDR method.

#### Brain microvessels preparation

Mouse brain microvessels were purified as previously described.^61^ Briefly, cerebral cortices from P11 mice were collected, homogenized, and centrifuged at 1,350 × g for 5 min at 4°C to remove debris. The resulting pellet was resuspended in 18% dextran prepared in Dulbecco’s PBS, vortexed for 2 min, and centrifuged at 6,080 × g for 10 min at 4°C. The final pellet, corresponding to the microvessel fraction, was resuspended in PBS containing protease inhibitors (cOmplete) and analyzed by Western blot.

#### BBB permeability studies

P11 mice were anesthetized with ketamine/xylazine and the thoracic cavity was opened surgically. A microsyringe, preloaded with 5 μL of a mixture of ^3^H- and ^14^C-labeled isotopes, was inserted into the apex of the heart, and the tracer mixture was rapidly injected as previously described.^3^ Ten seconds later, the right atrium was incised, and the animal was immediately perfused through the heart with 5 mL of ice-cold PBS. The brain and kidneys were then collected. The perfusion step ensured clearance of residual radioactive amino acids from the vascular compartment. A 15 sec arterial injection was previously used to monitor brain transport of amino acids.^62^ Brains and kidneys were homogenized in 1 mL of 0.1 M HCl, centrifuged at 16,000 × g for 10 min, and the resulting supernatant was transferred to scintillation vials containing 19 mL of Ultima Gold XR for scintillation counting. ^3^H radioactivity was measured within a 0–11.5 keV window, whereas ^14^C radioactivity was measured within a 35–156 keV window. The contribution of ^14^C spillover into the ^3^H channel was calculated for each sample and subtracted as background. The extraction fraction (EF) was defined as the ratio of amino acid counts per minute (CPM) detected in the brain to the total amino acid CPM injected. To account for differences in cardiac output, the EF of each amino acid was normalized to the EF of [^3^H]3-O-methyl glucose co-injected in the same experiment.

Fluorescein permeability was carried out in P11 mice as previously described.^3^ Fluorescein was intraperitoneally injected (10 mg/kg; Sigma-Aldrich, #F6377) and thirty minutes later, blood was collected from the right atrium. Mice were then transcardially perfused with 5 mL ice-cold PBS to remove residual intravascular fluorescein. Brains were harvested and homogenized in two volumes of 15% trichloroacetic acid (TCA), sonicated, and incubated on ice for 20 min, followed by centrifugation at 16,000 × g for 10 min. A 200-μL aliquot of the resulting supernatant was collected and alkalinized with 80 μL of 5 N NaOH. For serum analysis, blood samples were allowed to clot and then centrifuged at 8,000 × g for 5 min. Serum (20 μL) was mixed with 200 μL of 7.5% TCA, sonicated, and centrifuged at 16,000 × g for 10 min. A 200-μL aliquot of the supernatant was then collected and alkalinized with 40 μL of 5 N NaOH. Fluorescence intensity was measured using a microplate reader (Tecan Infinite M200 Pro) with excitation at 485 nm and emission at 530 nm. Standard curves were generated using 200-1000 ng fluorescein in 200 μL of 5 N NaOH. Brain fluorescein levels were normalized to serum fluorescein concentrations and expressed as (ng fluorescein/g wet tissue)/(ng/μL serum).

#### Transmission electron microscopy

For post-embedding immunogold labeling, P11 WT and Slc38a2-cKO mice were anesthetized and transcardially perfused with PBS containing 20 U/mL heparin, followed by fixation with 4% paraformaldehyde (PFA) and 0.1% glutaraldehyde (GA) in 0.1 M phosphate buffer (PB, pH 7.4). Brains were then removed, and the cerebral cortices were sectioned into 100-μm slices using a vibratome (Leica VT1200). Sections were collected in PBS and incubated in 0.5% sodium borohydride (NaBH_4_) in PBS for 30 min under gentle agitation. After four washes with 0.1 M PB, sections were rinsed in distilled water for 10 min and incubated in 2% aqueous uranyl acetate for 30 min at room temperature. Samples were subsequently dehydrated through a graded ethanol series and embedded in LR White resin according to the manufacturer’s instructions (Electron Microscopy Sciences). Ultrathin sections (75 nm) were then cut using a Leica UC7 ultramicrotome and collected on nickel grids. Sections were blocked in 1% bovine serum albumin (BSA) prepared in 40 mM Tris-HCl (pH 7.4) containing 300 mM NaCl. Grids were then incubated overnight at 4°C with anti-SLC38A2 antibody^53^ (4 μg/mL) diluted in the same buffer. After washing in 40 mM Tris-HCl (pH 7.4) supplemented with 300 mM NaCl, sections were incubated with a 12-nm colloidal gold-conjugated goat anti-rabbit secondary antibody diluted in 40 mM Tris-HCl (pH 7.4) containing 150 mM NaCl, 1% BSA, and 0.3% fish skin gelatin. As a negative control, *Slc38a2*-cKO sections were processed in parallel and incubated with anti-SLC38A2 under the same conditions. Following extensive washing, sections were counterstained with 2% uranyl acetate and examined using a Talos L120C transmission electron microscope operated at 120 kV.

For synaptic morphology analysis, P14 *Slc38a2*-cKO and WT mice (*n* = 3 per group) were perfused with 2% PFA and 2.5% GA in 0.1 M phosphate buffer (PB) and stored for 4 days at 4°C in 0.2% PFA and 0.25% GA in 0.1 M PB. Brains were then sectioned into 300-μm slices using a vibratome (Leica VT1200). Sections were post-fixed overnight at 4°C in 2% PFA and 2.5% GA prepared in 0.1 M sodium cacodylate buffer (pH 7.4), washed in the same buffer, and stained for 1 h on ice with 1% osmium tetroxide in 0.1 M sodium cacodylate buffer. Samples were subsequently stained *en bloc* with 1% uranyl acetate for 1 h on ice, dehydrated through a graded ethanol series, and embedded in Epon 812 resin. Ultrathin sections (75 nm) were cut from the ventral blade of the dentate gyrus molecular layer using a Leica UC7 ultramicrotome, mounted on copper grids, and examined with a Talos L120C transmission electron microscope operated at 120 kV. Asymmetric (excitatory) synapses were identified by the presence of a distinct synaptic cleft, a prominent postsynaptic density (PSD), and at least five synaptic vesicles in the presynaptic terminal. Twenty electron micrographs per mouse were acquired at 13,500× magnification. Symmetric synapses were identified by pre- and postsynaptic densities of comparable thickness. Quantitative analyses were performed by an investigator blinded to genotype.

#### Stable isotope tracing

Isotope tracing was carried out as previously described.^3^ For serine tracing, mice were intraperitoneally (*i.p.*) injected with Shin1 (50 mg/Kg), followed by three injections of a mixture of uniformly labeled glucose (glucose^13^C_6_, 1 g/Kg) and Ser^13^C_1_^15^N_1_ (50 mg/Kg) every 20 min. One hour after the last injection, the mice were anesthetized *i.p.* with ketamine (100 mg/kg) and xylazine (10 mg/kg). Blood was drawn from the right atrium, and the mice were transcardially perfused with 5 mL ice-cold phosphate-buffered saline (PBS). Blood was allowed to coagulate for 20 min, and sera were obtained by centrifugation at 8000 *x* g for 5 min. The brain regions were dissected, weighed, and stored at -80°C until metabolic profiling. Glutamine tracing was carried out by three injections of glutamine^13^C_5_^15^N_2_ (0.8 mmol/Kg) as described previously.^2^

#### Western blotting

Samples were homogenized by sonication in 40 mM Tris-HCl (pH 7.4), 100 mM NaCl, protein inhibitor cocktail (cOmplete Mini, Roche), and phosphatase inhibitor cocktail (PhosSTOP, Roche). Sample buffer containing SDS was added, and samples were heated and loaded on the 10% SDS-Page gels, and subsequently transferred to nitrocellulose membranes. For SLC38A2 immunodetection, the samples were loaded without heating. Antibody sources are listed in the key resources table.

#### Motor behavior

All behavioral assessments were performed with the investigator blinded to genotype. Grip strength was evaluated by placing mice on an iron grid that was gradually tilted from horizontal to vertical, and the angle at which the mice lost grip was recorded. In the hindlimb suspension test, mice were suspended by their hindlimbs in padded 50-mL conical tubes, and the latency to fall was measured. In the forelimb suspension test, pups were allowed to grasp a wire stretched across a padded metal cup with both forepaws, and the latency to fall was recorded. For the negative geotaxis test, pups were placed head-down on an inclined surface, and the time required to reorient and turn 180° was measured. For each assay, the mean of three trials was used for analysis.^63^

### Quantification and statistical analysis

Data are expressed as mean ± SEM, and graphs were generated using GraphPad Prism 8.2.1. For comparisons among multiple groups, one-way ANOVA was followed by Holm-Sidak’s multiple comparisons test for post hoc analysis. Comparisons between two groups were analyzed using unpaired two-tailed Student’s *t*-tests or Mann-Whitney when the distribution was not normal. FDR was calculated by the method of Benjamini, Krieger and Yekutieli. All statistical analyses are described in the figure legends. The cutoff for statistical significance was *p* <0.05.

## Supplemental information

Document S1. Figures S1–S7

Table S1. Metabolomics of cortex, hippocampus, liver, and kidneys

Table S2. Sphingolipidomics of hippocampus, liver, kidneys, and serum

Table S3. Differentially expressed genes in the cortex of mice fed with -SG and CD

Table S4. Metabolomics of cortex, hippocampus, and serum of P11 WT and Slc38a2-cKO mice

Table S5. Differentially expressed genes in the hippocampus of P11 WT and Slc38a2-cKO mice

Table S6. Metabolomics of the hippocampus of P28 and P84 WT and Slc38a2-cKO mice

## References

[1] Odeh M., Sajrawi C., Majcher A., Zubedat S., Shaulov L., Radzishevsky A., Mizrahi L., Chung W.K., Avital A., Hornemann T. (2024). A new type of blood-brain barrier aminoacidopathy underlies metabolic microcephaly associated with SLC1A4 mutations. Brain.

[2] Radzishevsky I., Harb L., Odeh M., Maoz I., Saeed A., Lahkar A., Agranovich B., Abramovich I., Chaudhry F.A., Avital A. (2026). SLC38A3 deficiency reveals a critical role of blood-derived glutamine in brain development. Brain.

[3] Radzishevsky I., Odeh M., Bodner O., Zubedat S., Shaulov L., Litvak M., Esaki K., Yoshikawa T., Agranovich B., Li W.H. (2023). Impairment of serine transport across the blood-brain barrier by deletion of Slc38a5 causes developmental delay and motor dysfunction. Proc. Natl. Acad. Sci. USA.

[4] Tărlungeanu D.C., Deliu E., Dotter C.P., Kara M., Janiesch P.C., Scalise M., Galluccio M., Tesulov M., Morelli E., Sonmez F.M. (2016). Impaired Amino Acid Transport at the Blood Brain Barrier Is a Cause of Autism Spectrum Disorder. Cell.

[5] Sweeney M.D., Zhao Z., Montagne A., Nelson A.R., Zlokovic B.V. (2019). Blood-Brain Barrier: From Physiology to Disease and Back. Physiol. Rev..

[6] Damseh N., Simonin A., Jalas C., Picoraro J.A., Shaag A., Cho M.T., Yaacov B., Neidich J., Al-Ashhab M., Juusola J. (2015). Mutations in SLC1A4, encoding the brain serine transporter, are associated with developmental delay, microcephaly and hypomyelination. J. Med. Genet..

[7] Marafi D., Fatih J.M., Kaiyrzhanov R., Ferla M.P., Gijavanekar C., Al-Maraghi A., Liu N., Sites E., Alsaif H.S., Al-Owain M. (2022). Biallelic variants in SLC38A3 encoding a glutamine transporter cause epileptic encephalopathy. Brain.

[8] Yang J.H., Wada A., Yoshida K., Miyoshi Y., Sayano T., Esaki K., Kinoshita M.O., Tomonaga S., Azuma N., Watanabe M. (2010). Brain-specific Phgdh deletion reveals a pivotal role for L-serine biosynthesis in controlling the level of D-serine, an N-methyl-D-aspartate receptor co-agonist, in adult brain. J. Biol. Chem..

[9] Lone M.A., Santos T., Alecu I., Silva L.C., Hornemann T. (2019). 1-Deoxysphingolipids. Biochim. Biophys. Acta Mol. Cell Biol. Lipids.

[10] Majcher A., Karsai G., Yusifov E., Schaettin M., Malagola E., Horvath P., Li J., Rodriguez-Gallardo S., Shimizu K., Zhibo G. (2025). Very long-chain fatty acids drive 1-deoxySphingolipid toxicity. Nat. Commun..

[11] Hatanaka T., Huang W., Wang H., Sugawara M., Prasad P.D., Leibach F.H., Ganapathy V. (2000). Primary structure, functional characteristics and tissue expression pattern of human ATA2, a subtype of amino acid transport system A. Biochim. Biophys. Acta.

[12] Yao D., Mackenzie B., Ming H., Varoqui H., Zhu H., Hediger M.A., Erickson J.D. (2000). A novel system A isoform mediating Na+/neutral amino acid cotransport. J. Biol. Chem..

[13] Reimer R.J., Chaudhry F.A., Gray A.T., Edwards R.H. (2000). Amino acid transport system A resembles system N in sequence but differs in mechanism. Proc. Natl. Acad. Sci. USA.

[14] Gaccioli F., Huang C.C., Wang C., Bevilacqua E., Franchi-Gazzola R., Gazzola G.C., Bussolati O., Snider M.D., Hatzoglou M. (2006). Amino acid starvation induces the SNAT2 neutral amino acid transporter by a mechanism that involves eukaryotic initiation factor 2alpha phosphorylation and cap-independent translation. J. Biol. Chem..

[15] Hoffmann T.M., Cwiklinski E., Shah D.S., Stretton C., Hyde R., Taylor P.M., Hundal H.S. (2018). Effects of Sodium and Amino Acid Substrate Availability upon the Expression and Stability of the SNAT2 (SLC38A2) Amino Acid Transporter. Front. Pharmacol..

[16] Palii S.S., Thiaville M.M., Pan Y.X., Zhong C., Kilberg M.S. (2006). Characterization of the amino acid response element within the human sodium-coupled neutral amino acid transporter 2 (SNAT2) System A transporter gene. Biochem. J..

[17] Hyde R., Christie G.R., Litherland G.J., Hajduch E., Taylor P.M., Hundal H.S. (2001). Subcellular localization and adaptive up-regulation of the System A (SAT2) amino acid transporter in skeletal-muscle cells and adipocytes. Biochem. J..

[18] Hyde R., Peyrollier K., Hundal H.S. (2002). Insulin promotes the cell surface recruitment of the SAT2/ATA2 system A amino acid transporter from an endosomal compartment in skeletal muscle cells. J. Biol. Chem..

[19] Uno K., Yamada T., Ishigaki Y., Imai J., Hasegawa Y., Sawada S., Kaneko K., Ono H., Asano T., Oka Y., Katagiri H. (2015). A hepatic amino acid/mTOR/S6K-dependent signalling pathway modulates systemic lipid metabolism via neuronal signals. Nat. Commun..

[20] Jenstad M., Quazi A.Z., Zilberter M., Haglerød C., Berghuis P., Saddique N., Goiny M., Buntup D., Davanger S., S Haug F.M. (2009). System A transporter SAT2 mediates replenishment of dendritic glutamate pools controlling retrograde signaling by glutamate. Cereb. Cortex.

[21] Mackenzie B., Erickson J.D. (2004). Sodium-coupled neutral amino acid (System N/A) transporters of the SLC38 gene family. Pflugers Arch.

[22] Grewal S., Defamie N., Zhang X., De Gois S., Shawki A., Mackenzie B., Chen C., Varoqui H., Erickson J.D. (2009). SNAT2 amino acid transporter is regulated by amino acids of the SLC6 gamma-aminobutyric acid transporter subfamily in neocortical neurons and may play no role in delivering glutamine for glutamatergic transmission. J. Biol. Chem..

[23] Zhou X., Azimi M., Handin N., Riselli A., Vora B., Chun E., Dang Z., Huang E., Yee S.W., Artursson P., Giacomini K.M. (2025). Proteomic profiling reveals age-related changes in transporter proteins in the human blood-brain barrier. Sci. Rep..

[24] Rabattoni V., Marchesani F., Murtas G., Sacchi S., Mozzarelli A., Bruno S., Peracchi A., Pollegioni L., Campanini B. (2023). The human phosphorylated pathway: a multienzyme metabolic assembly for l-serine biosynthesis. FEBS J..

[25] Wolosker H. (2018). The Neurobiology of d-Serine Signaling. Adv. Pharmacol..

[26] Alecu I., Tedeschi A., Behler N., Wunderling K., Lamberz C., Lauterbach M.A.R., Gaebler A., Ernst D., Van Veldhoven P.P., Al-Amoudi A. (2017). Localization of 1-deoxysphingolipids to mitochondria induces mitochondrial dysfunction. J. Lipid Res..

[27] Bodner O., Radzishevsky I., Foltyn V.N., Touitou A., Valenta A.C., Rangel I.F., Panizzutti R., Kennedy R.T., Billard J.M., Wolosker H. (2020). D-Serine Signaling and NMDAR-Mediated Synaptic Plasticity Are Regulated by System A-Type of Glutamine/D-Serine Dual Transporters. J. Neurosci..

[28] Zhang Y., Chen K., Sloan S.A., Bennett M.L., Scholze A.R., O'Keeffe S., Phatnani H.P., Guarnieri P., Caneda C., Ruderisch N. (2014). An RNA-sequencing transcriptome and splicing database of glia, neurons, and vascular cells of the cerebral cortex. J. Neurosci..

[29] Edwards R.H. (2007). The neurotransmitter cycle and quantal size. Neuron.

[30] Sánchez del Pino M.M., Peterson D.R., Hawkins R.A. (1995). Neutral amino acid transport characterization of isolated luminal and abluminal membranes of the blood-brain barrier. J. Biol. Chem..

[31] Ducker G.S., Ghergurovich J.M., Mainolfi N., Suri V., Jeong S.K., Hsin-Jung Li S., Friedman A., Manfredi M.G., Gitai Z., Kim H., Rabinowitz J.D. (2017). Human SHMT inhibitors reveal defective glycine import as a targetable metabolic vulnerability of diffuse large B-cell lymphoma. Proc. Natl. Acad. Sci. USA.

[32] Furuya S., Tabata T., Mitoma J., Yamada K., Yamasaki M., Makino A., Yamamoto T., Watanabe M., Kano M., Hirabayashi Y. (2000). L-serine and glycine serve as major astroglia-derived trophic factors for cerebellar Purkinje neurons. Proc. Natl. Acad. Sci. USA.

[33] Park D.K., Petshow S., Anisimova M., Barragan E.V., Gray J.A., Stein I.S., Zito K. (2022). Reduced d-serine levels drive enhanced non-ionotropic NMDA receptor signaling and destabilization of dendritic spines in a mouse model for studying schizophrenia. Neurobiol. Dis..

[34] Diniz L.P., Almeida J.C., Tortelli V., Vargas Lopes C., Setti-Perdigão P., Stipursky J., Kahn S.A., Romão L.F., de Miranda J., Alves-Leon S.V. (2012). Astrocyte-induced synaptogenesis is mediated by transforming growth factor beta signaling through modulation of D-serine levels in cerebral cortex neurons. J. Biol. Chem..

[35] Crain B., Cotman C., Taylor D., Lynch G. (1973). A quantitative electron microscopic study of synaptogenesis in the dentate gyrus of the rat. Brain Res..

[36] Horio M., Kohno M., Fujita Y., Ishima T., Inoue R., Mori H., Hashimoto K. (2011). Levels of D-serine in the brain and peripheral organs of serine racemase (Srr) knock-out mice. Neurochem. Int..

[37] Son H., Kim S., Jung D.H., Baek J.H., Lee D.H., Roh G.S., Kang S.S., Cho G.J., Choi W.S., Lee D.K., Kim H.J. (2019). Insufficient glutamine synthetase activity during synaptogenesis causes spatial memory impairment in adult mice. Sci. Rep..

[38] Wolosker H., Blackshaw S., Snyder S.H. (1999). Serine racemase: a glial enzyme synthesizing D-serine to regulate glutamate-N-methyl-D-aspartate neurotransmission. Proc. Natl. Acad. Sci. USA.

[39] Liu Y., Wu Z., Armstrong D.W., Wolosker H., Zheng Y. (2023). Detection and analysis of chiral molecules as disease biomarkers. Nat. Rev. Chem.

[40] Souza I.N.d.O., Roychaudhuri R., de Belleroche J., Mothet J.P. (2023). d-Amino acids: new clinical pathways for brain diseases. Trends Mol. Med..

[41] Henneberger C., Papouin T., Oliet S.H.R., Rusakov D.A. (2010). Long-term potentiation depends on release of D-serine from astrocytes. Nature.

[42] Dallérac G., Li X., Lecouflet P., Morisot N., Sacchi S., Asselot R., Pham T.H., Potier B., Watson D.J.G., Schmidt S. (2021). Dopaminergic neuromodulation of prefrontal cortex activity requires the NMDA receptor coagonist d-serine. Proc. Natl. Acad. Sci. USA.

[43] Neame S., Safory H., Radzishevsky I., Touitou A., Marchesani F., Marchetti M., Kellner S., Berlin S., Foltyn V.N., Engelender S. (2019). The NMDA receptor activation by d-serine and glycine is controlled by an astrocytic Phgdh-dependent serine shuttle. Proc. Natl. Acad. Sci. USA.

[44] Perez E.J., Tapanes S.A., Loris Z.B., Balu D.T., Sick T.J., Coyle J.T., Liebl D.J. (2017). Enhanced astrocytic d-serine underlies synaptic damage after traumatic brain injury. J. Clin. Investig..

[45] Sasabe J., Chiba T., Yamada M., Okamoto K., Nishimoto I., Matsuoka M., Aiso S. (2007). D-serine is a key determinant of glutamate toxicity in amyotrophic lateral sclerosis. EMBO J..

[46] Wong J.M., Folorunso O.O., Barragan E.V., Berciu C., Harvey T.L., Coyle J.T., Balu D.T., Gray J.A. (2020). Postsynaptic Serine Racemase Regulates NMDA Receptor Function. J. Neurosci..

[47] Burnet P.W.J., Hutchinson L., von Hesling M., Gilbert E.J., Brandon N.J., Rutter A.R., Hutson P.H., Harrison P.J. (2008). Expression of D-serine and glycine transporters in the prefrontal cortex and cerebellum in schizophrenia. Schizophr. Res..

[48] Pernot P., Maucler C., Tholance Y., Vasylieva N., Debilly G., Pollegioni L., Cespuglio R., Marinesco S. (2012). d-Serine diffusion through the blood-brain barrier: effect on d-serine compartmentalization and storage. Neurochem. Int..

[49] Lee W.J., Hawkins R.A., Viña J.R., Peterson D.R. (1998). Glutamine transport by the blood-brain barrier: a possible mechanism for nitrogen removal. Am. J. Physiol..

[50] Wang Y., Zhou L., Wang N., Qiu B., Yao D., Yu J., He M., Li T., Xie Y., Yu X. (2025). Comprehensive characterization of metabolic consumption and production by the human brain. Neuron.

[51] Pérez-Arellano I., Carmona-Álvarez F., Martínez A.I., Rodríguez-Díaz J., Cervera J. (2010). Pyrroline-5-carboxylate synthase and proline biosynthesis: from osmotolerance to rare metabolic disease. Protein Sci..

[52] Norenberg M.D., Rao K.V.R., Jayakumar A.R. (2005). Mechanisms of ammonia-induced astrocyte swelling. Metab. Brain Dis..

[53] Takanaga H., Tokuda N., Ohtsuki S., Hosoya K.I., Terasaki T. (2002). ATA2 is predominantly expressed as system A at the blood-brain barrier and acts as brain-to-blood efflux transport for L-proline. Mol. Pharmacol..

[54] Du C., Xu H., Zhao W., Jiao L., Chen Y., Sun X., Cao M., Zhang Y., Guo Y., Qiao R. (2025). Inhibiting SLC38A2 lowers blood pressure in rodent models of hypertension. Sci. Transl. Med..

[55] Wolosker H., Liebl D.J. (2026). A mitochondrial-neuroinflammation-D-serine connection in epilepsy. Proc. Natl. Acad. Sci. USA.

[56] Jiang J., Zuo M., Zhao K., Ling Z., Wu Z., Xue D., Mo S., Liu Y., Chen Y., Wang J. (2026). mtDNA leakage promotes neuron-glia crosstalk to induce epilepsy by cGAS-STING-driven neuroinflammation and serine metabolic reprogramming. Proc. Natl. Acad. Sci. USA.

[57] Tapanes S.A., Arizanovska D., Díaz M.M., Folorunso O.O., Harvey T., Brown S.E., Radzishevsky I., Close L.N., Jagid J.R., Graciolli Cordeiro J. (2022). Inhibition of glial D-serine release rescues synaptic damage after brain injury. Glia.

[58] Scott A.J., Mittal A., Meghdadi B., O’Brien A., Bailleul J., Sravya P., Achreja A., Zhou W., Xu J., Lin A. (2025). Rewiring of cortical glucose metabolism fuels human brain cancer growth. Nature.

[59] Kumar S., Wang C.Y., Lalwani H.K., Ngadio J.L., Wulandari F.S., Solomon D.D., Liu H.P. (2026). Comprehensive In Silico Investigation of L-Glutamine Transporters and Metabolism in Glioblastoma. Pharmaceuticals.

[60] Mackay G.M., Zheng L., van den Broek N.J.F., Gottlieb E. (2015). Analysis of Cell Metabolism Using LC-MS and Isotope Tracers. Methods Enzymol..

[61] Assmann J., Müller K., Wenzel J., Walther T., Brands J., Thornton P., Allan S., Schwaninger M. (2017). Isolation and Cultivation of Primary Brain Endothelial Cells from Adult Mice. Bio. Protoc..

[62] Oldendorf W.H. (1971). Brain uptake of radiolabeled amino acids, amines, and hexoses after arterial injection. Am. J. Physiol..

[63] Feather-Schussler D.N., Ferguson T.S. (2016). A Battery of Motor Tests in a Neonatal Mouse Model of Cerebral Palsy. J. Vis. Exp..

