# Supplementary material for: Endothelial SLC38A2 controls brain D-serine homeostasis and synapse development
Source: iScience. 2026 Sep 9;29(10):117487. doi: 10.1016/j.isci.2026.117487 (PMC13583979; doi:10.1016/j.isci.2026.117487)
Supplement: Supplementary file 1 [file mmc1.pdf]

iScience, Volume *29*

Supplemental information

Endothelial SLC38A2 controls brain D-serine homeostasis and synapse development

Akshay K. Tiwari, Ankita Lahkar, Clara Sajrawi, Yara Hussein, Adam Majcher, Stephane Bonelli, Bella Agranovich, Ifat Abramovich, Shani Stern, Farrukh A. Chaudhry, Thorsten Hornemann, Inna Radzishevsky, and Herman Wolosker

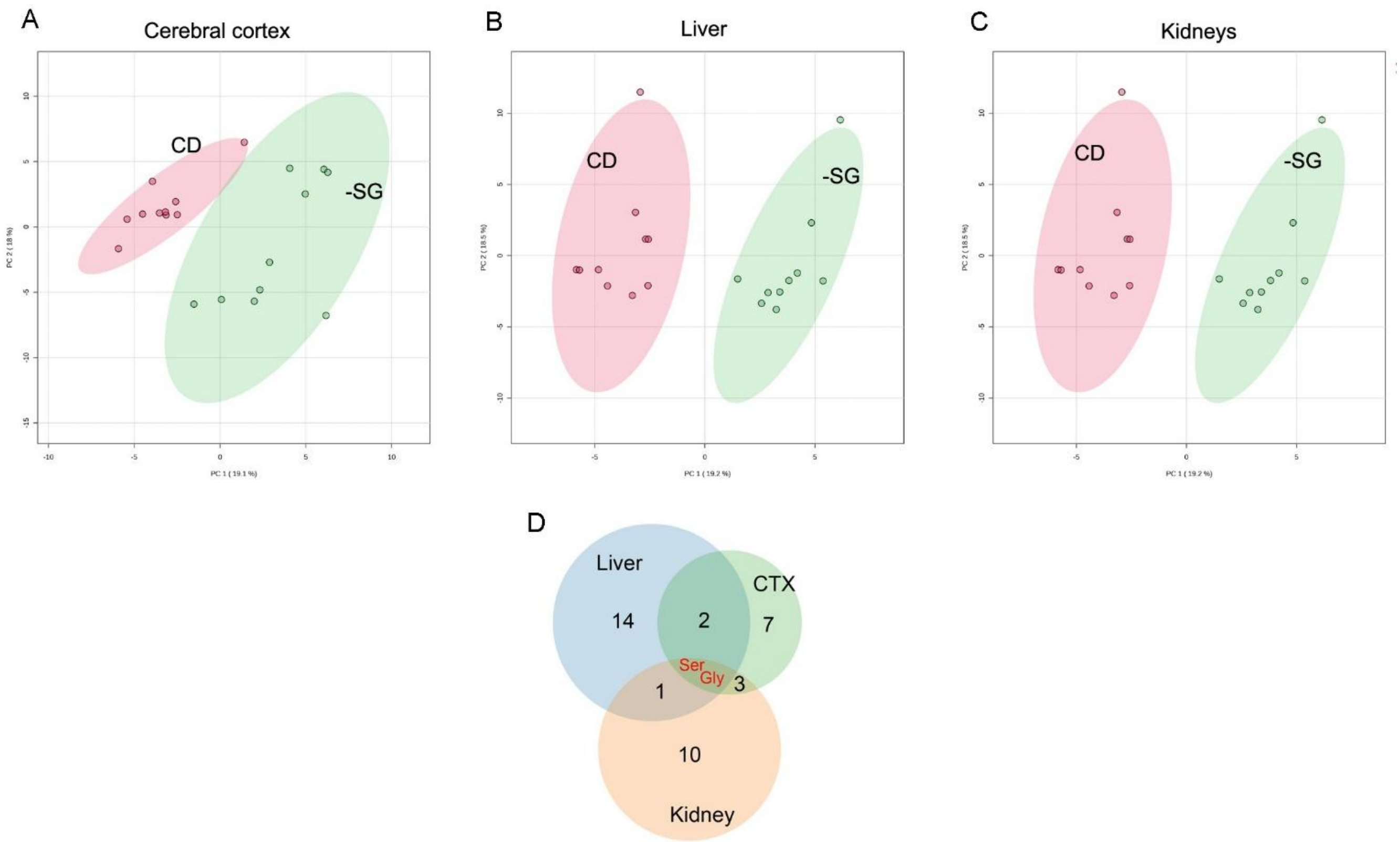

Fig S1

Principal component analysis (PCA) and Venn diagram of -SG vs CD-fed mice

(A) PCA analysis of the metabolome in the cerebral cortices of -SG vs CD-fed mice (n=10 mice/group).

(B) PCA analysis of the metabolome in livers of -SG vs CD-fed mice (n=10 mice/group).

(C) PCA analysis of the metabolome in kidneys of -SG vs CD-fed mice (n=10 mice/group).

(E) Venn diagram of changed metabolites in the cerebral cortex, liver, and kidneys of -SG-fed mice.

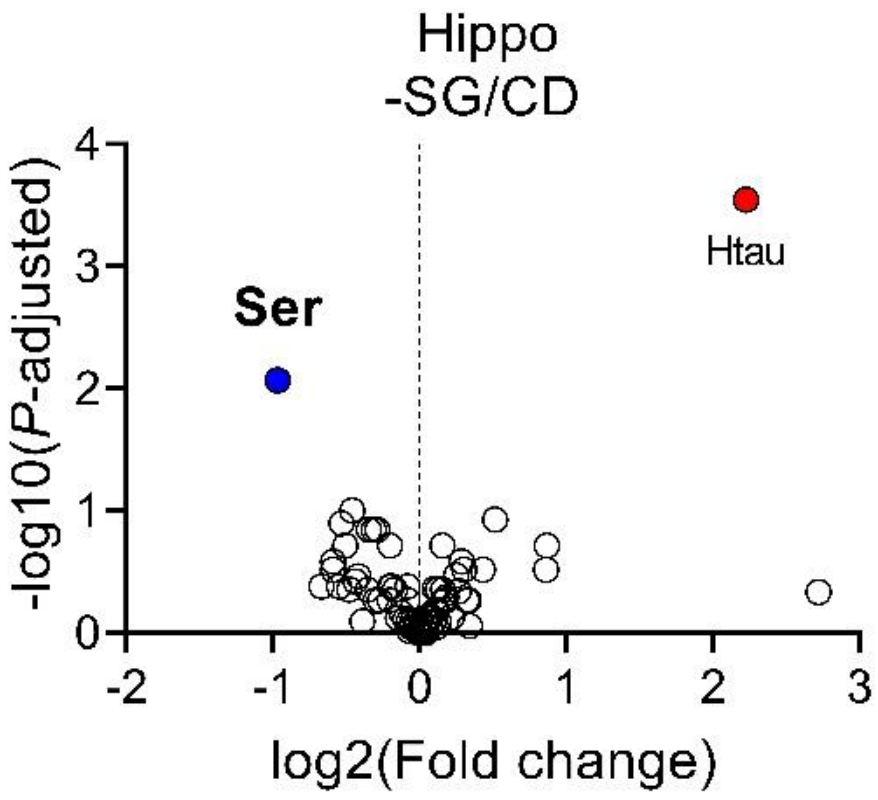

Fig S2

Volcano analysis of the metabolome in the hippocampus of -SG vs CD-fed mice.

The data were obtained from 4-week-old mice fed with either CD (5 mice) or -SG (6 mice) diet. Significantly changed metabolites having an FDR < 0.05 are depicted in color. Htau, hypotaurine.

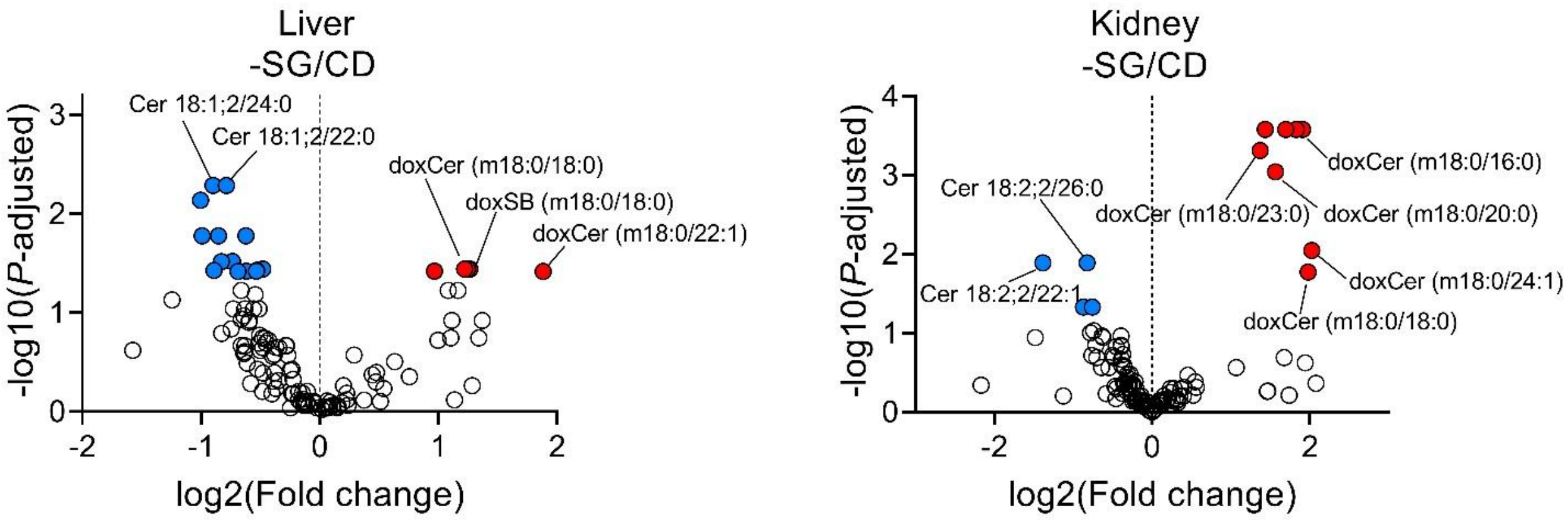

Fig S3

Volcano analysis of sphingolipids in liver and kidneys of -SG-fed mice

(A) Significantly changed sphingolipids in the liver of -SG-fed mice compared to CD (n=10 mice/group). Significantly changed metabolites having an FDR < 0.05 are depicted in color.

(B) Significantly changed sphingolipids in the kidneys of -SG-fed mice compared to CD (n=10 mice/group). Significantly changed metabolites having an FDR < 0.05 are depicted in color.

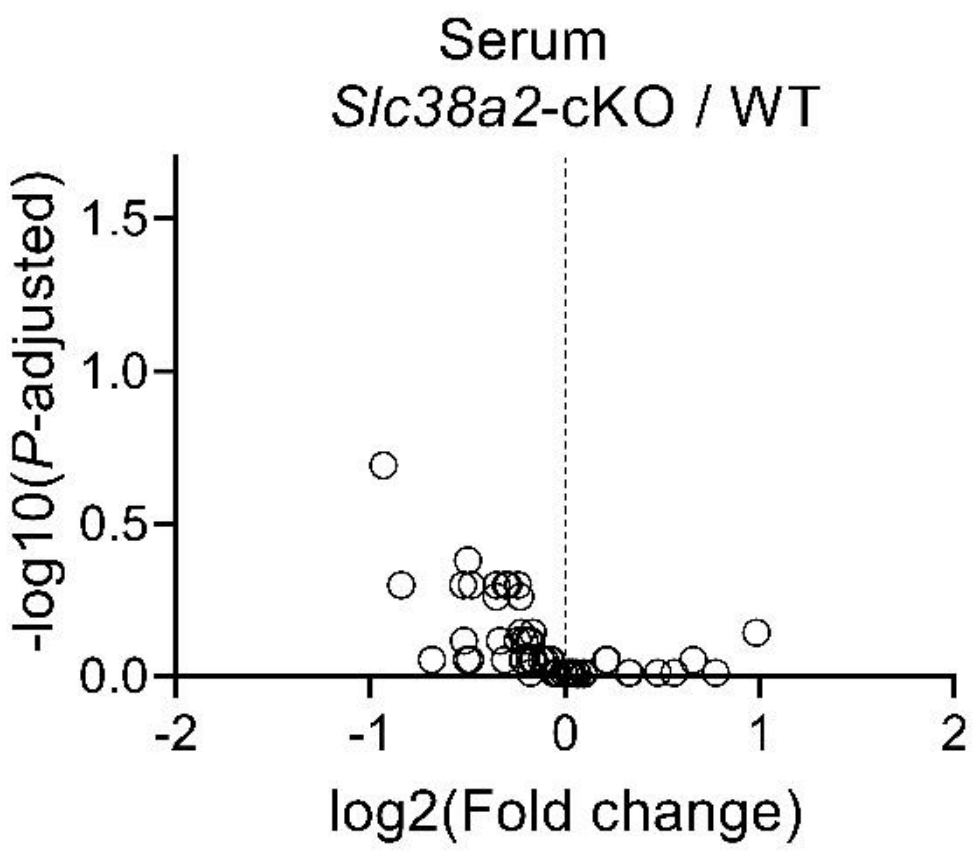

Fig S4

Volcano plot analysis of the serum metabolites in P11 WT and *Slc38a2*-cKO pups. N= 8 mice/group.

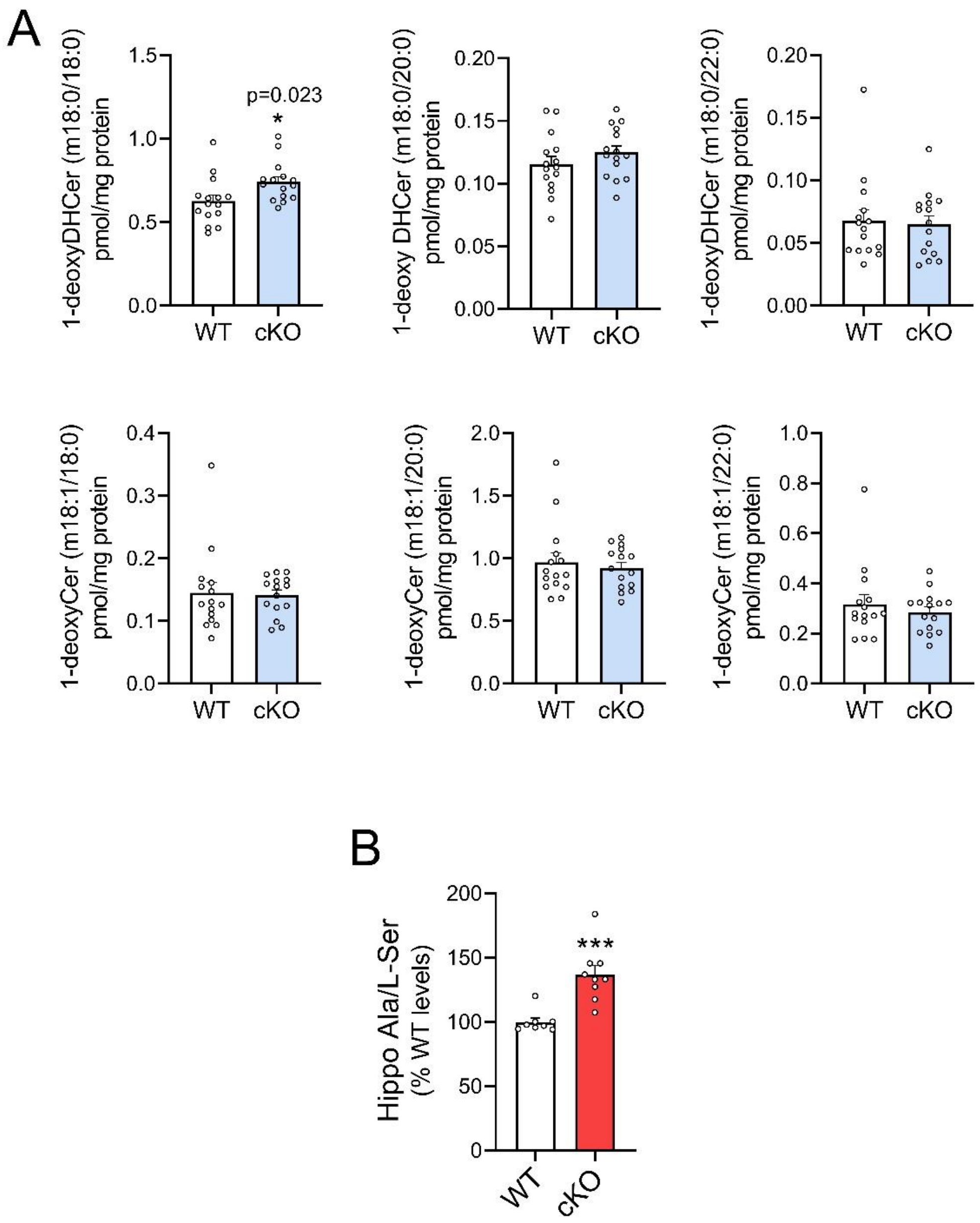

Fig S5

Hippocampal levels of selected deoxydihydroceramides and Ala/Ser ratio in *Slc38a2*-cKO pups.

(A) Hippocampal levels of selected 1-deoxydihydroceramides (m18:0/x:x) and 1-deoxyceramides (m18:1/x:x) in P11 *Slc38a2*-cKO compared with WT littermates. The data are mean ± S.E.M. of 15 mice/group. Student's t test.

(B) Ratio of hippocampal alanine to total serine in *Slc38a2*-cKO compared with WT littermates. The data are mean ± S.E.M. of 8-9 mice/group. Student's t test.

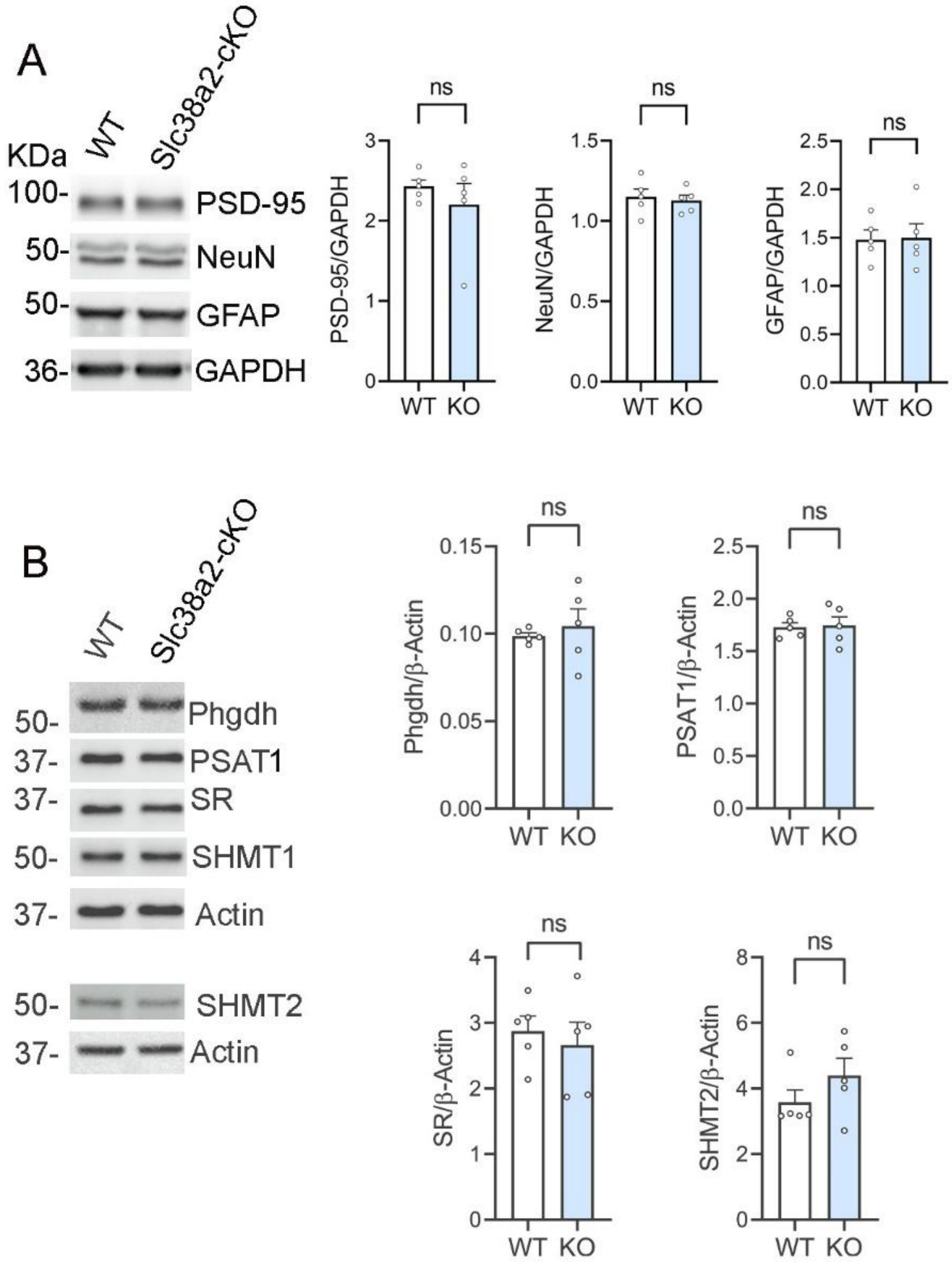

Fig S6

Expression of neuronal and glial markers and serine metabolic enzymes in P11 *Slc38a2*-cKO pups. Representative Western blots of the hippocampus, along with the quantitative expression normalized by GAPDH or actin (n=5 mice/group); Student's t test. Phgdh, 3-phosphoglygerate dehydrogenase; PSAT1, Phosphoserine aminotransferase; SHMT, serine hydroxymethyltransferase; SR, serine racemase.

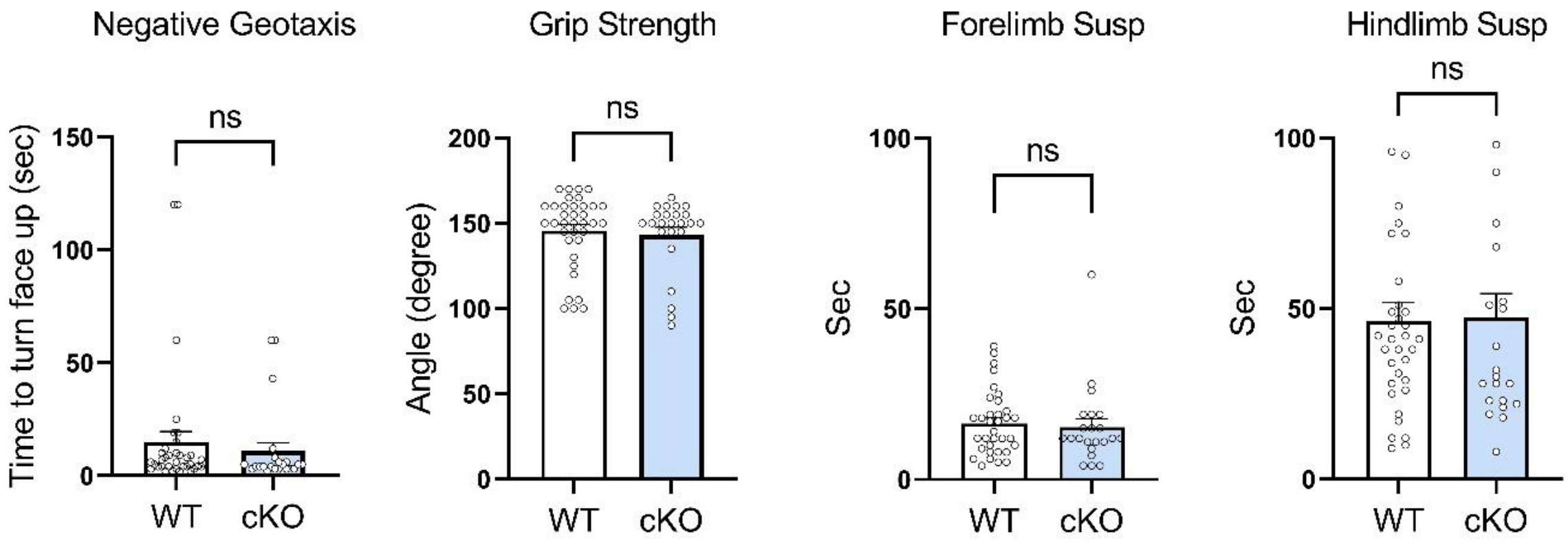

Fig S7

Motor behavior is unaltered in Slc38a2-cKO pups.

(A) Negative geotaxis was assessed by measuring the time required for pups to rotate 180° after being placed in a head-down position.

(B) Grip strength was assessed by measuring the angle at which pups fell from a tilted wire-grid surface.

(C) Upper limb suspension test, in which mice are suspended by their forelimbs.

(D) Hindlimb suspension test, measured as the latency to fall while suspended by the hindlimbs.

Data in A-D are means ± SEM of 36 (WT) and 23 (Slc38a2-cKO) mice/group. Mann-Whitney test.

Data S1- Raw uncropped images of Western blot membranes

Fig 2B

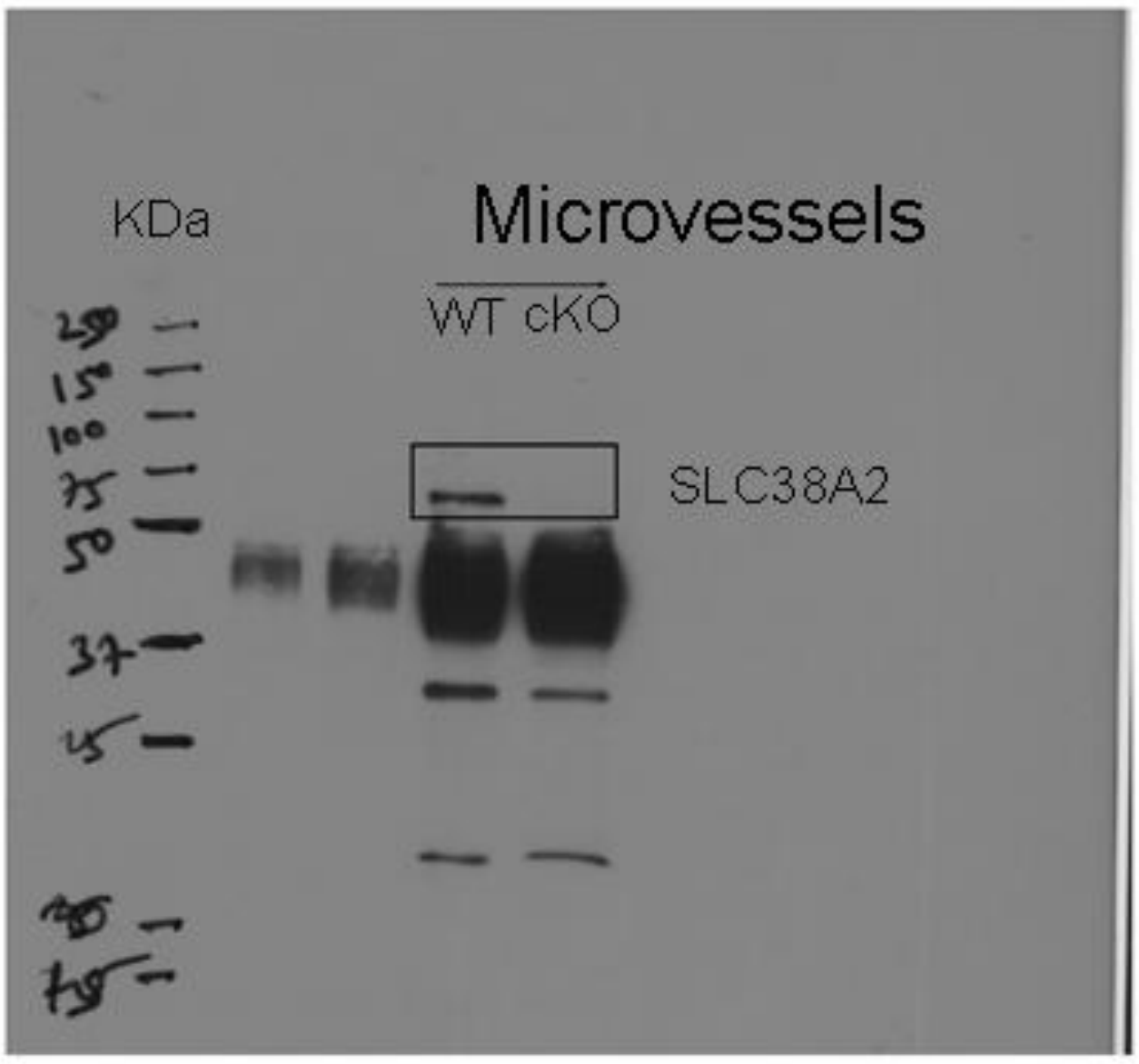

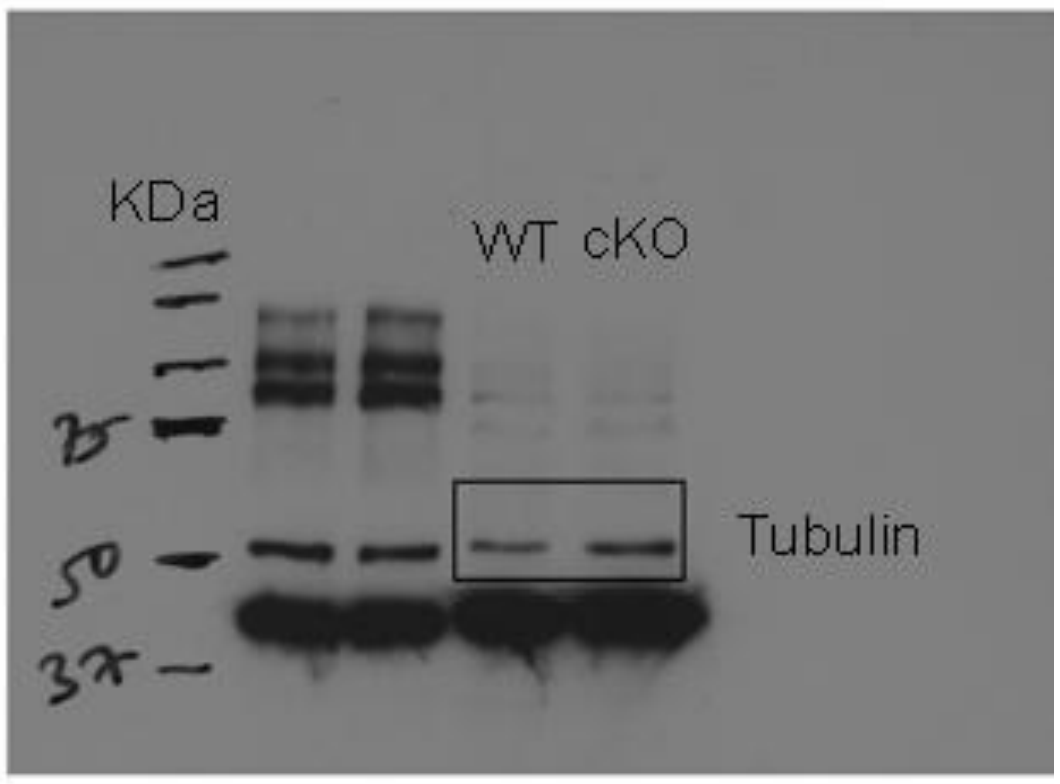

Fig S6A

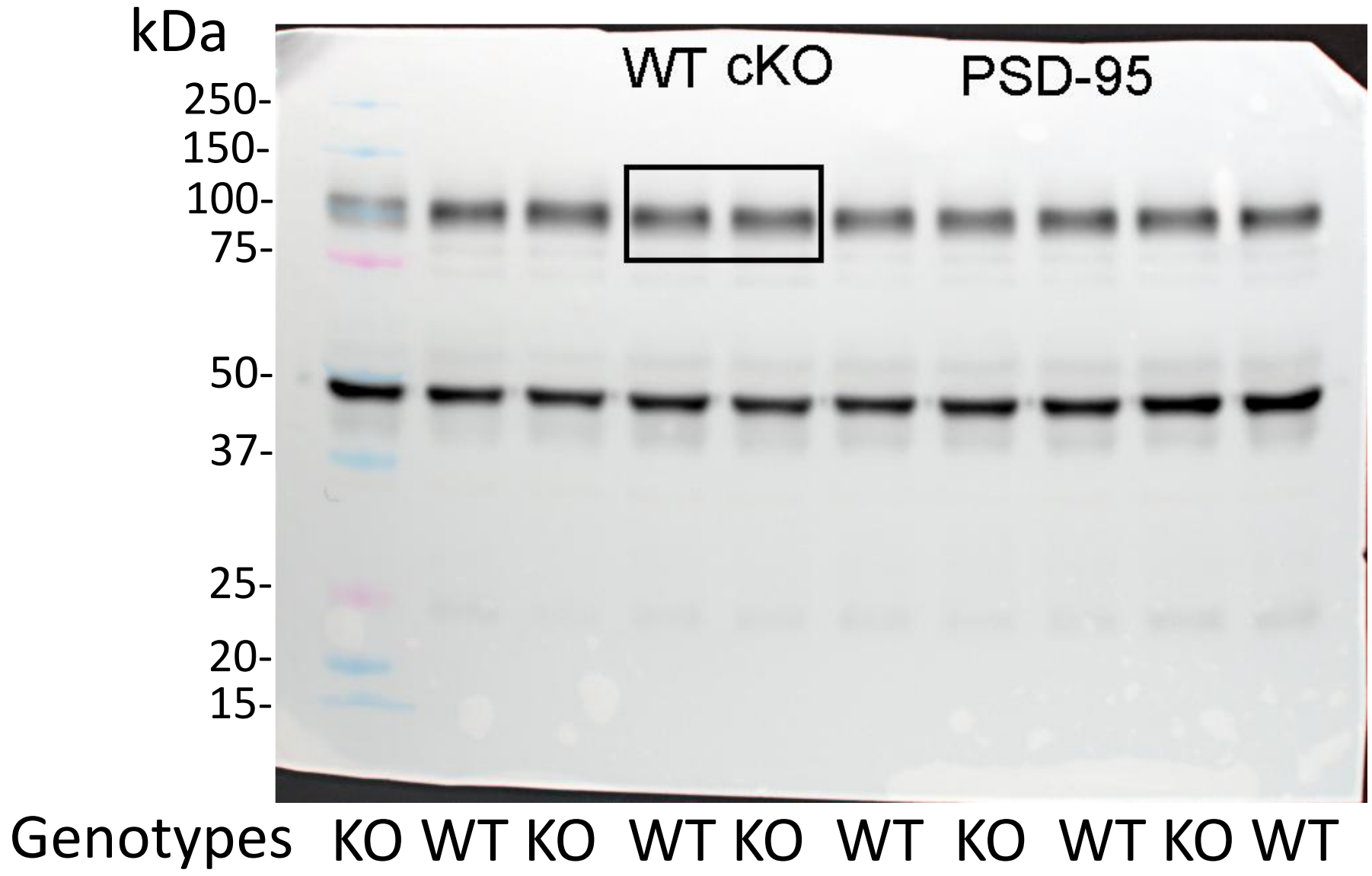

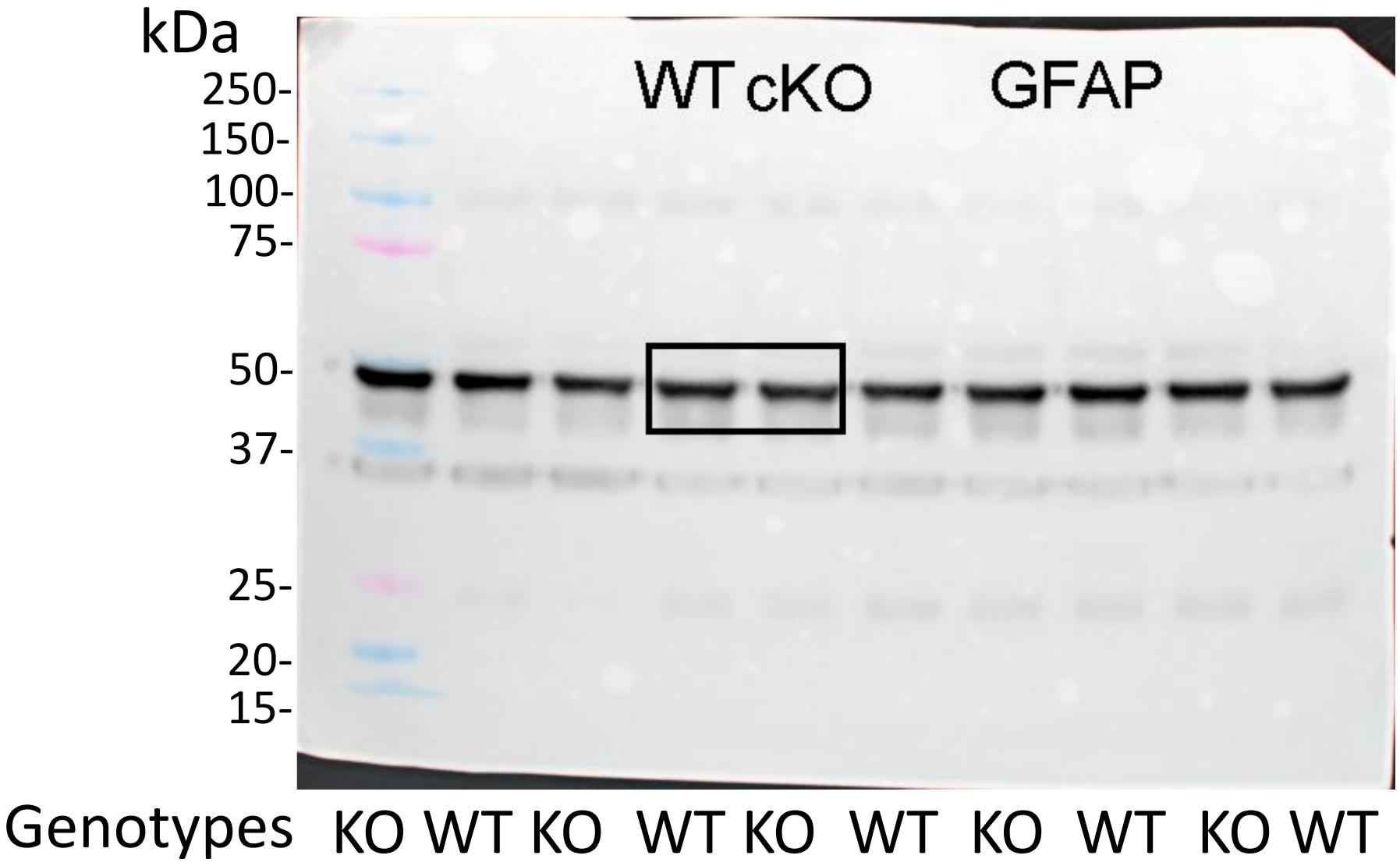

Fig S6A

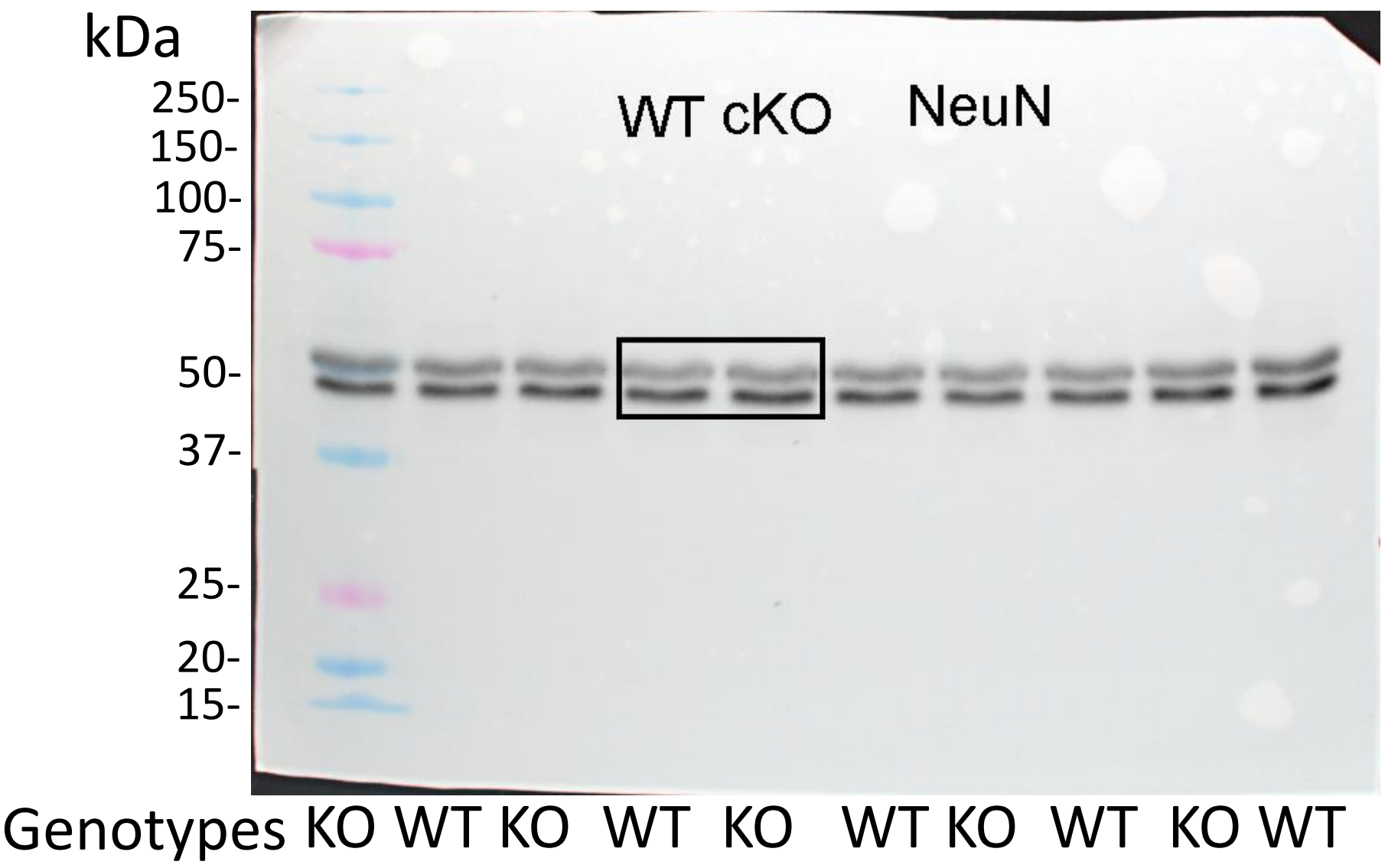
kDa
250-
150-
100-
75-
50-
37-
25-
20-
15-
WT cKO
NeuN
Genotypes KO WT KO WT KO WT KO WT KO WT

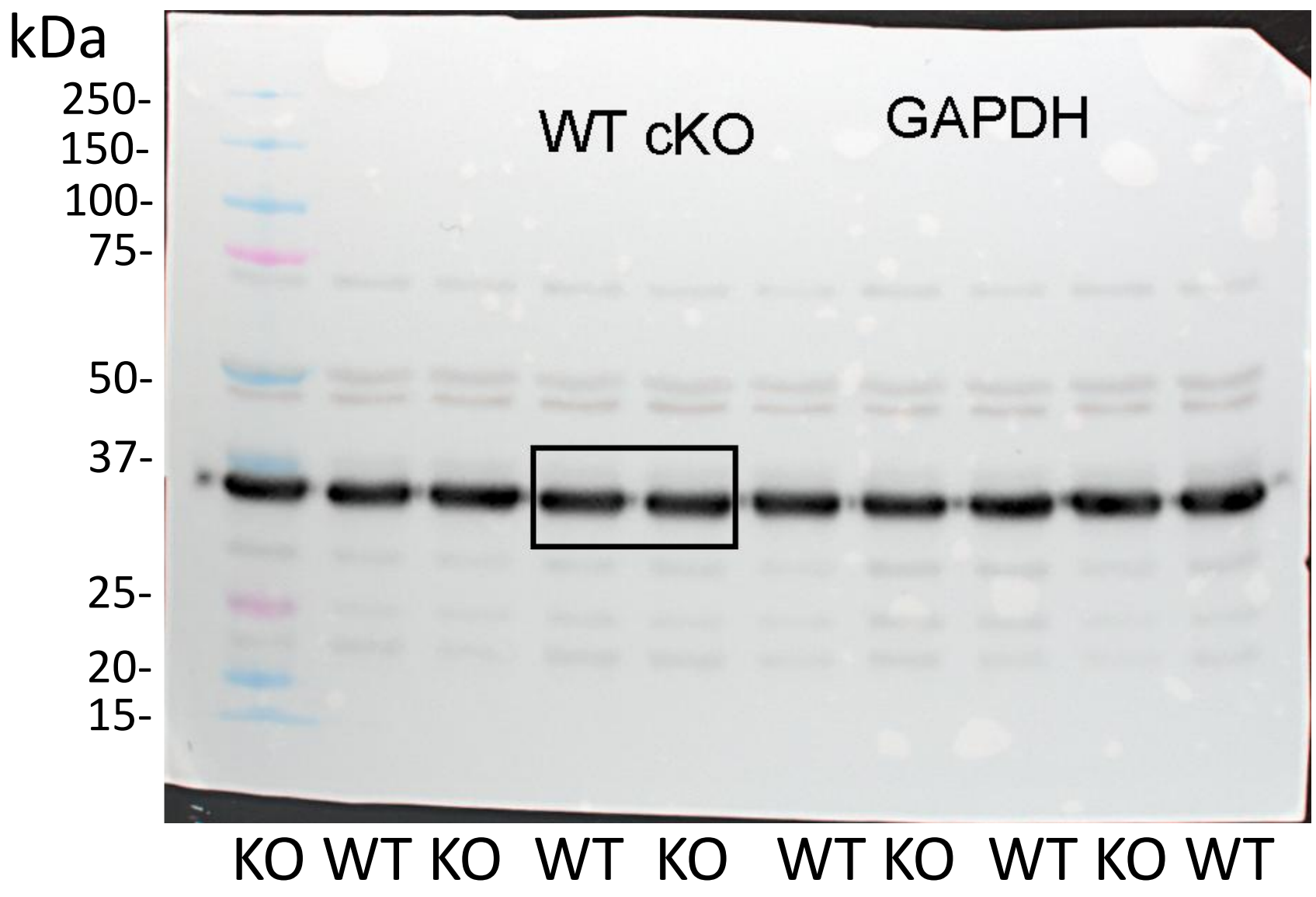
kDa
250-
150-
100-
75-
50-
37-
25-
20-
15-
WT cKO
GAPDH
KO WT KO WT KO WT KO WT KO WT

Fig S6B

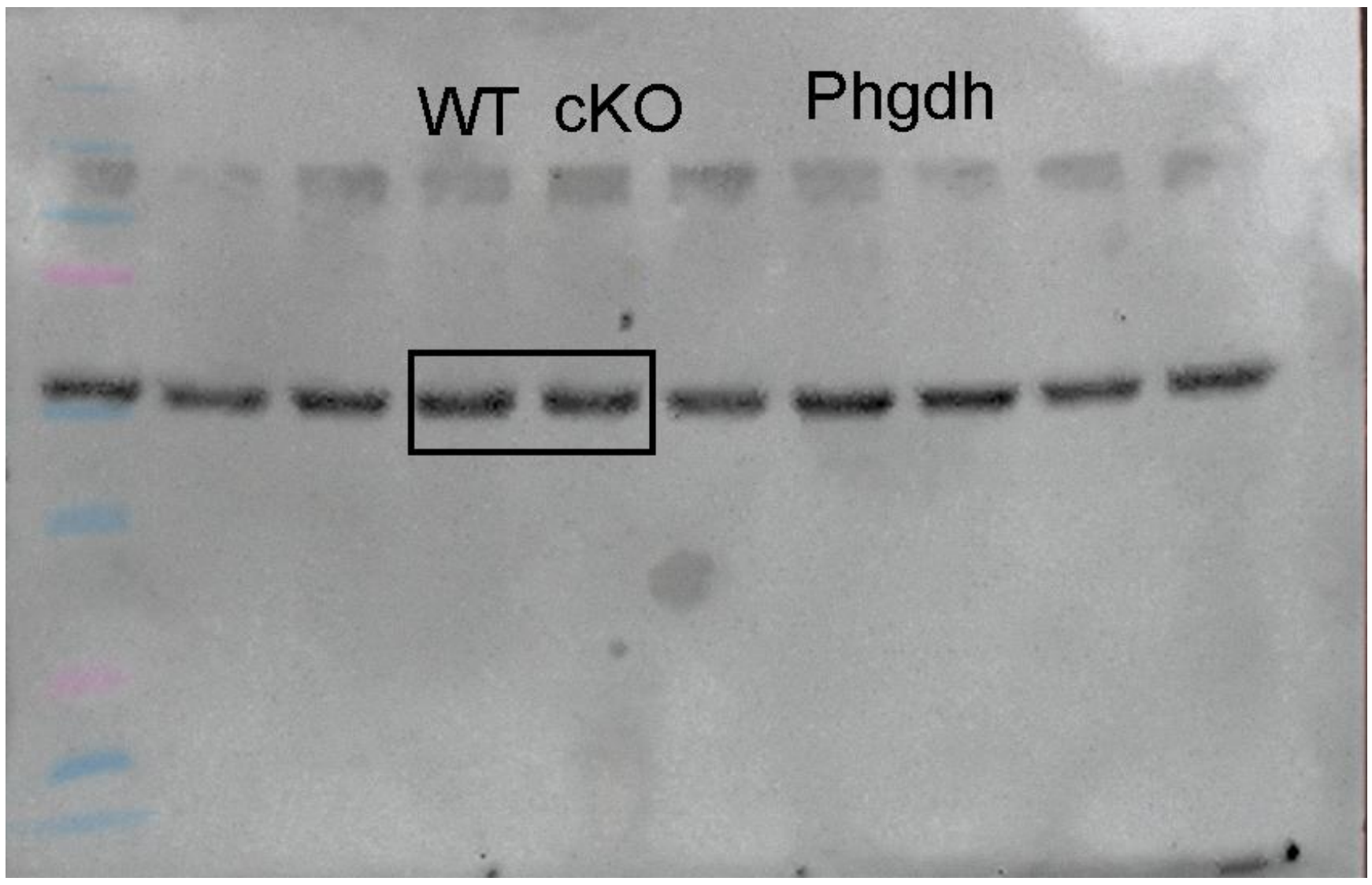
kDa
250-
150-
100-
75-
50-
37-
25-
20-
15-
WT cKO
Phgdh
Genotypes KO WT KO WT KO WT KO WT KO WT

kDa

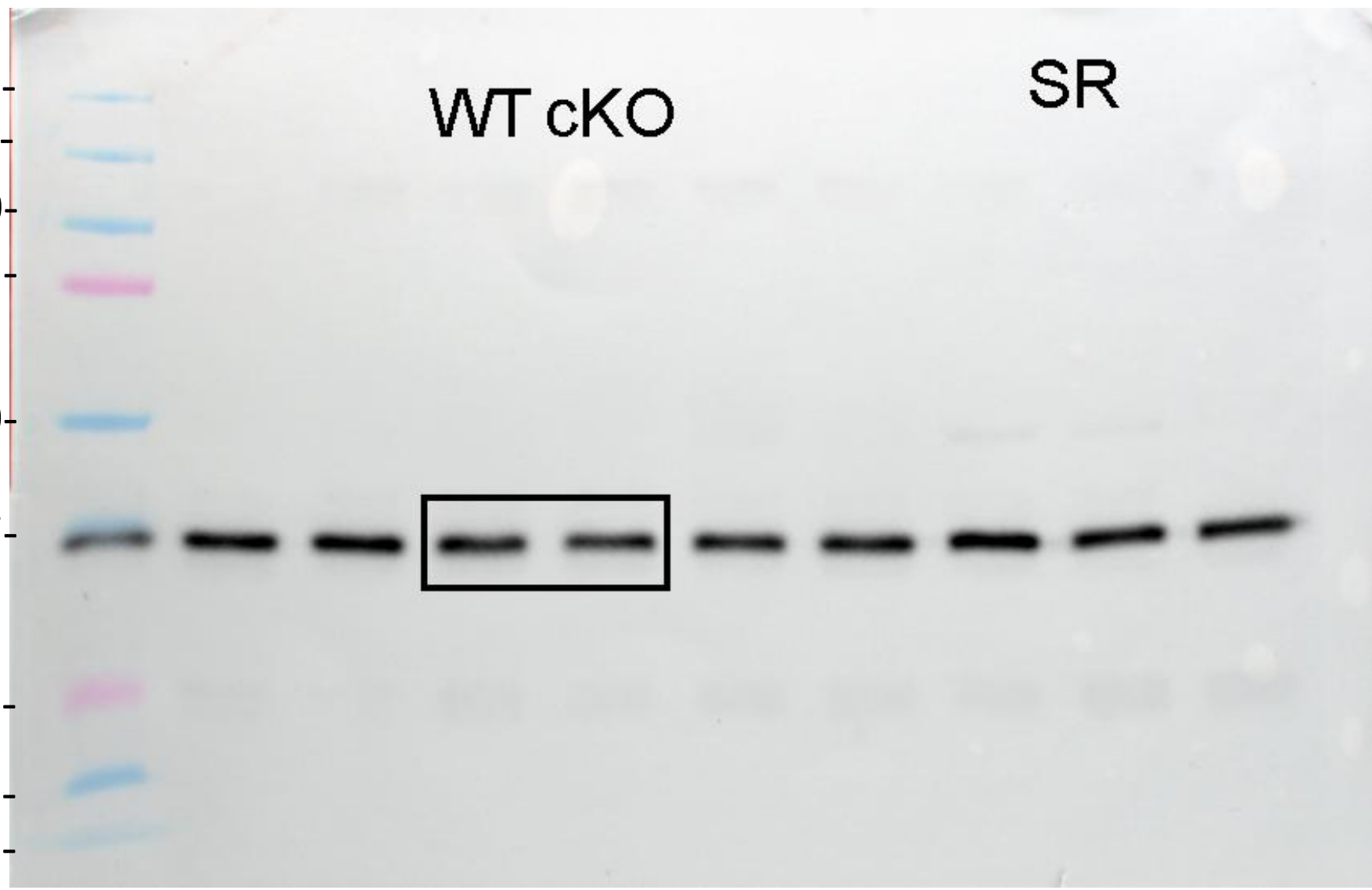
250-
150-
100-
75-
50-
37-
25-
20-
15-
WT cKO
SR
Genotypes KO WT KO WT KO WT KO WT KO WT

Fig S6B

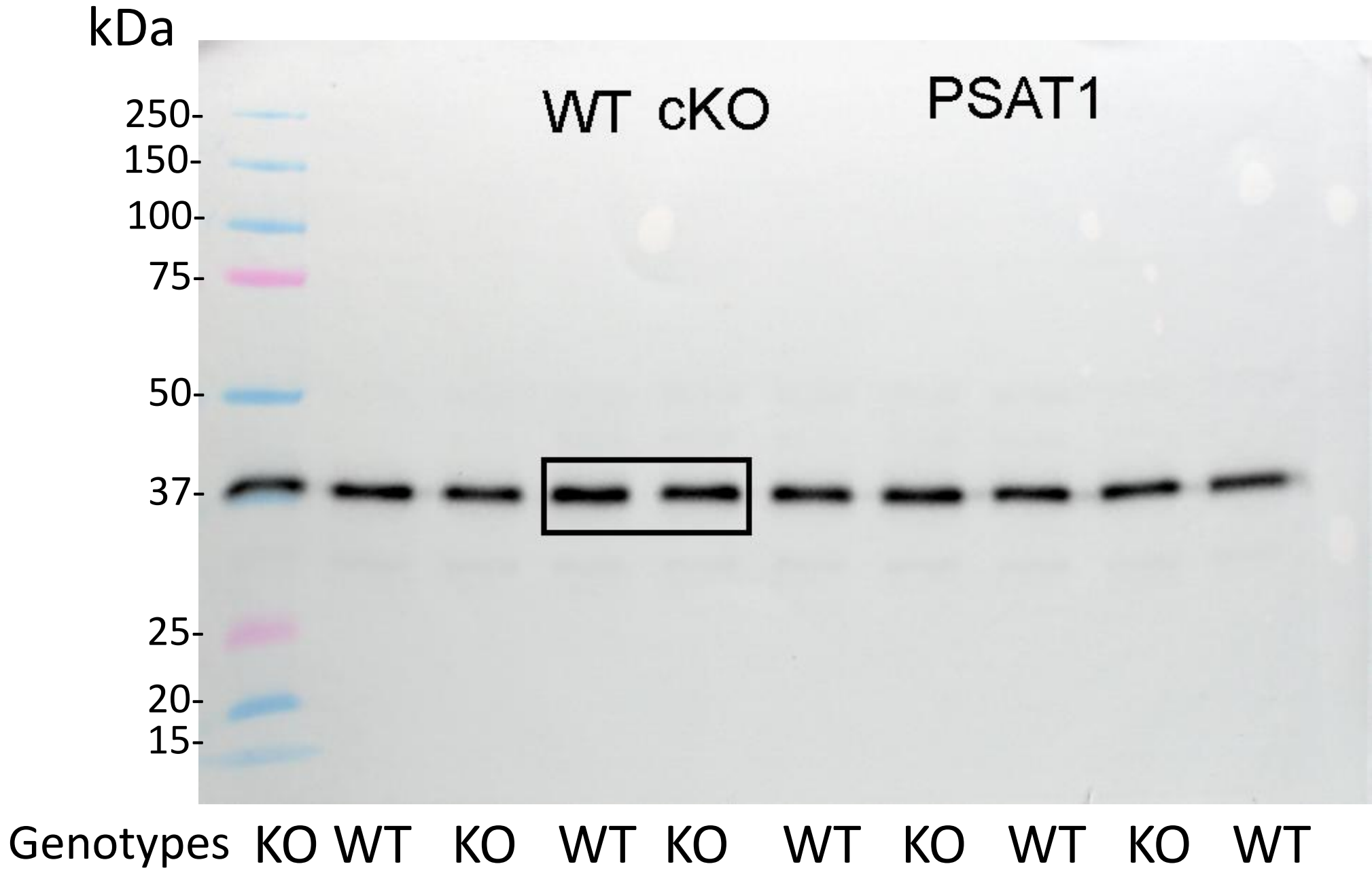
kDa
250-
150-
100-
75-
50-
37-
25-
20-
15-
WT cKO
PSAT1
Genotypes KO WT KO WT KO WT KO WT KO WT

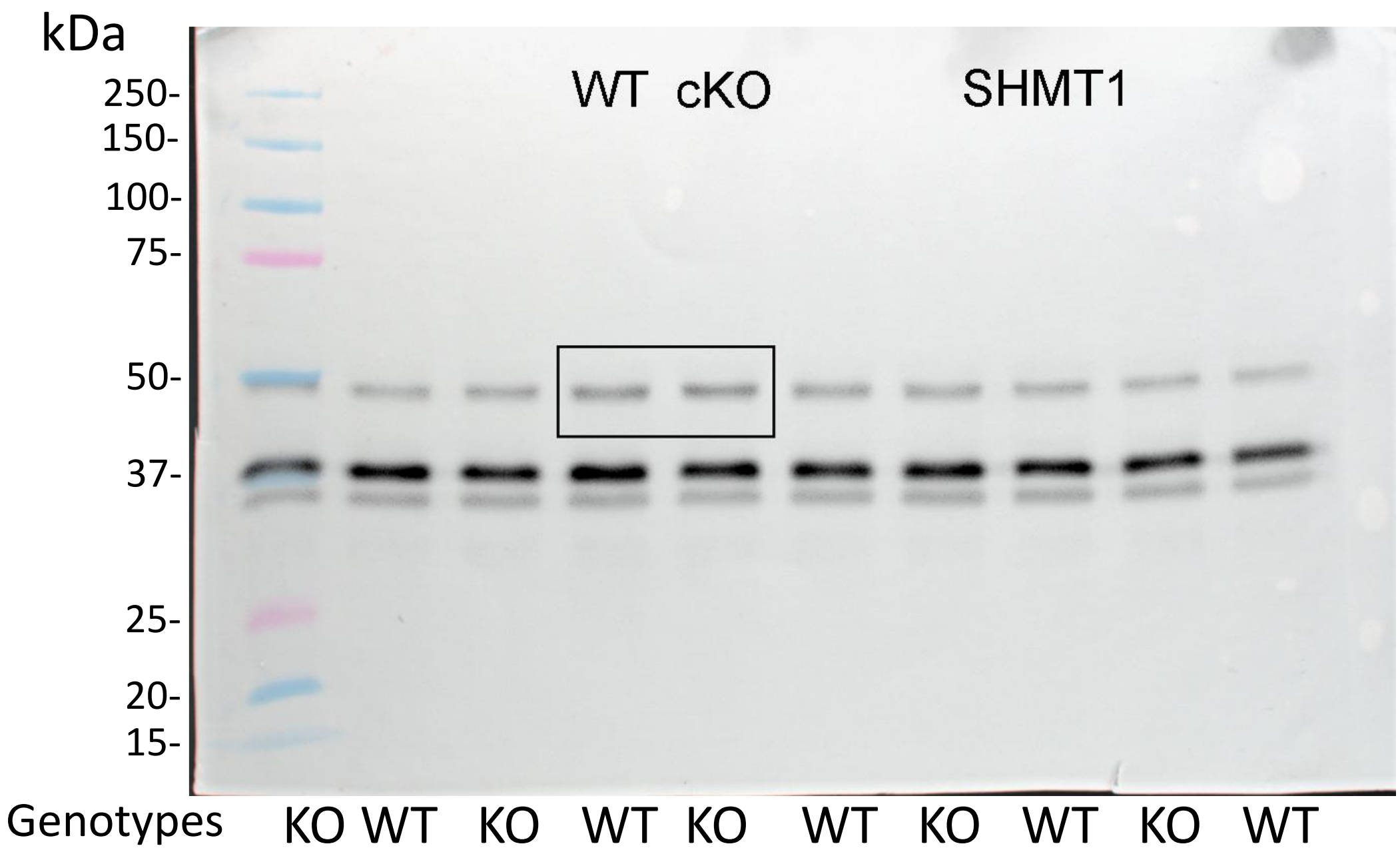
kDa
250-
150-
100-
75-
50-
37-
25-
20-
15-
WT cKO
SHMT1
Genotypes KO WT KO WT KO WT KO WT KO WT

Fig S6B

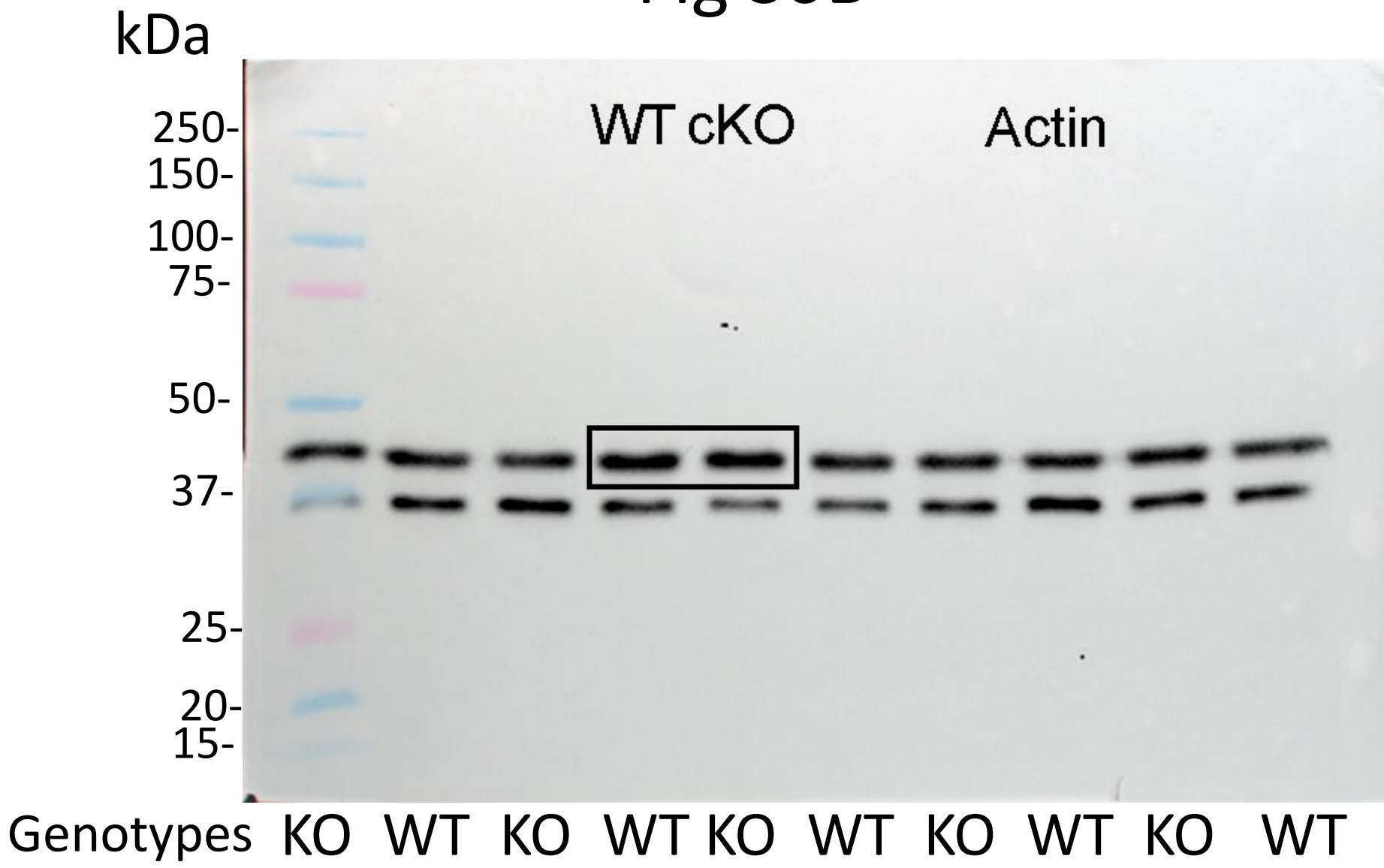
kDa
250-
150-
100-
75-
50-
37-
25-
20-
15-
WT cKO
Actin
Genotypes KO WT KO WT KO WT KO WT KO WT

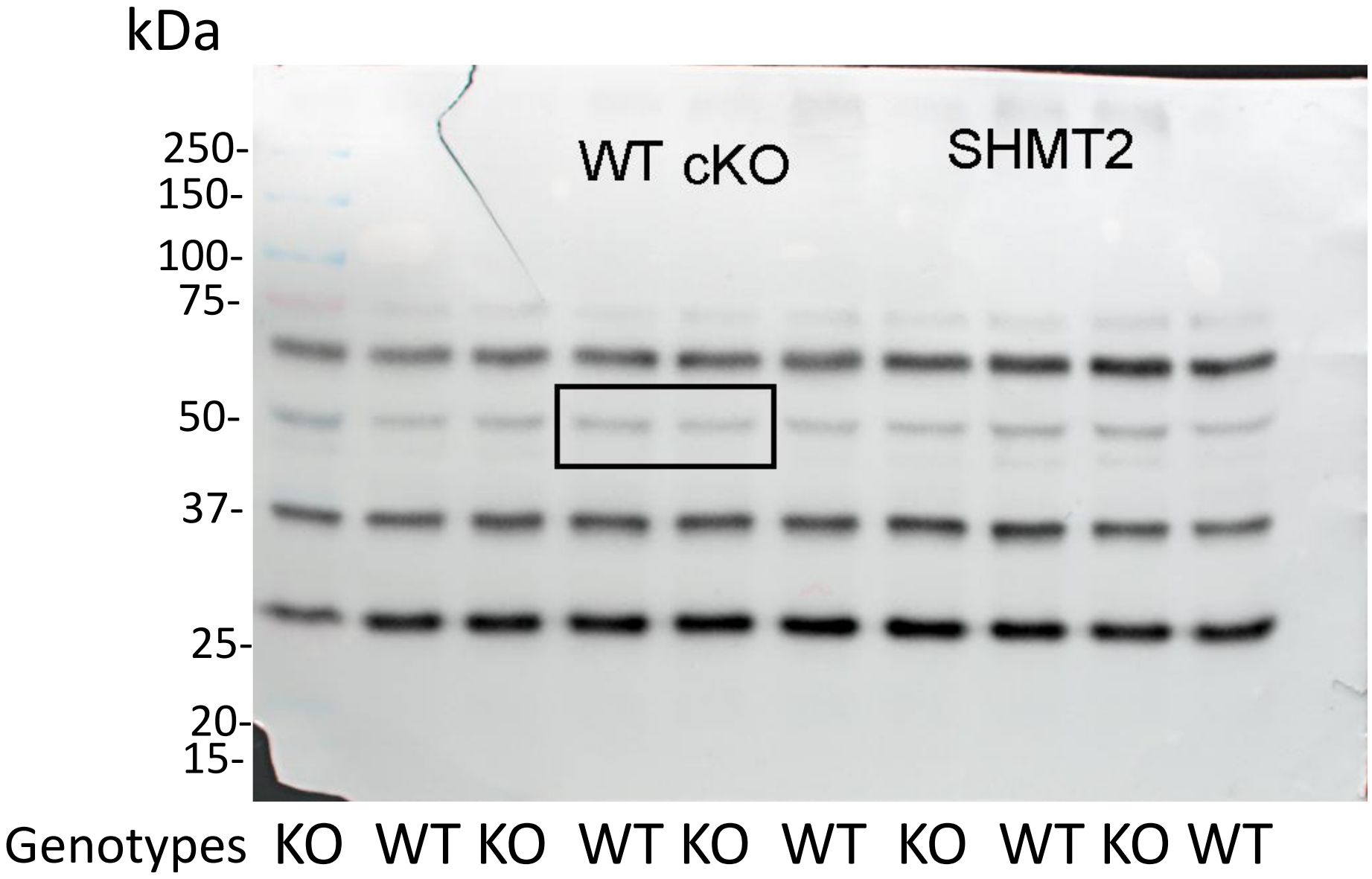
kDa
250-
150-
100-
75-
50-
37-
25-
20-
15-
WT cKO
SHMT2
Genotypes KO WT KO WT KO WT KO WT KO WT

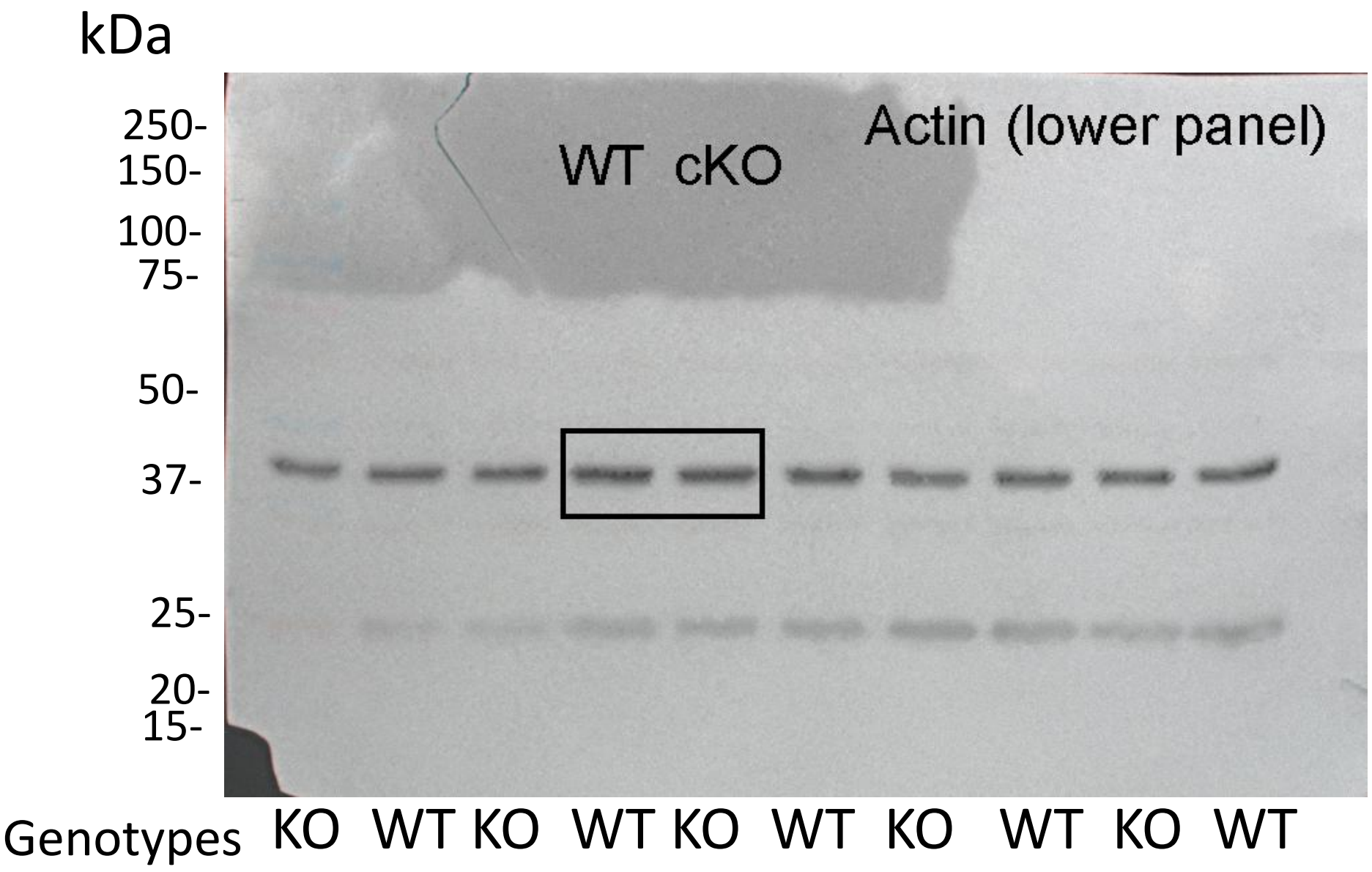
kDa
250-
150-
100-
75-
50-
37-
25-
20-
15-
WT cKO
Actin (lower panel)
Genotypes KO WT KO WT KO WT KO WT KO WT
